# Multi objective optimization and evaluation approach of prefabricated component combination solutions using NSGA-II and simulated annealing optimized projection pursuit method

**DOI:** 10.1038/s41598-024-65319-3

**Published:** 2024-07-19

**Authors:** Qun Wang, Xizhen Xu, Xiaoxin Ding, Tiebing Chen, Ronghui Deng, Jinglei Li, Jiawei Jiang

**Affiliations:** 1 School of Construction Engineering, Shenzhen Polytechnic University, Shenzhen, 518055 China; 2 School of Economics and Management, Jilin Jianzhu University, Changchun, 130119 China; 3 Zhongnan University of Economics and Law, Wuhan, 430073 China

**Keywords:** Engineering, Civil engineering

## Abstract

As a main carrier mode for the sustainable development of the construction industry in China, prefabricated building may lead to problems such as cost overruns, project delays, and waste of resources due to unreasonable selection of prefabricated components. Therefore, we quantitatively analyze the contribution rate of quality optimization of prefabricated components using QFD-SEM. Under the constraints of prefabrication rate, quality optimization contribution rate, and expected values of various sub-goals, we propose a multi-objective optimization method for prefabricated component combinations based on cost, duration, and carbon emissions. By using NSGA-II to solve the model, we can obtain a set of optimal Pareto solutions for prefabricated component combinations. Based on the optimal Pareto solution set, we establish a multi-objective evaluation model using simulated annealing optimization projection tracing method, and select the optimal prefabricated component combination solution according to the projected eigenvalues of the solutions. An empirical study is conducted using an eleven-story framed building in Shenzhen, Guangdong Province, China as a case study. The results show that: (1) Using this method, optimal solutions can be obtained in an unbounded solution space, with the optimal solution having advantages over both fully cast-in-place and fully prefabricated solutions. Compared to the fully cast-in-place solution, the duration and carbon emissions are reduced by 36.62% and 12.74% respectively, while compared to the fully prefabricated solution, costs are reduced by 4.15%. (2) There is a certain negative correlation between the cost of prefabricated component combinations and duration, carbon emissions, and quality optimization, while there is a certain positive correlation with the prefabrication rate. (3) The size of the optimal projection direction vector based on the optimization objectives indicates that carbon emissions have the greatest impact on the evaluation results of the solutions.

## Introduction

With the rapid development of urbanization, the role of the construction industry as a pillar industry of the national economy has been continuously enhanced. During the “13th Five-Year Plan” period, China's construction industry has achieved significant results in reform and development, with an average annual growth rate of 5.1% in the national construction industry value-added, accounting for over 6.9% of the GDP^1^. According to the “Analysis of Development Statistics in the Construction Industry in the First Half of 2023”, the construction industry grew by 7.7% year-on-year in the first half of 2023, with a growth rate 2.2 percentage points higher than the GDP growth rate. However, the construction industry in China has long been developed in an extensive mode, resulting in problems such as low efficiency, waste of resources, and inadequate management level^2^. At the same time, the rapid growth of industrialization has posed severe threats and challenges to global ecological resources, and various industries have begun to recognize the importance of sustainable development^3^. In 2020, the total carbon emissions from the entire process of construction in China reached 5.08 billion tons of CO_2_, accounting for 50.9% of the country's total carbon emissions. This reflects the significant potential for emission reduction in the construction industry^4^. By adopting reasonable and moderate per capita construction area, a carbon emissions reduction of 58% can be achieved in the construction material production stage. By adopting passive building design and applying efficient HVAC systems, a 25% reduction in building carbon emissions can be achieved. The application of digitalization and renewable energy technologies can further reduce carbon emissions by 7%^5^. As one of the high-energy-consuming industries, the construction industry has made energy conservation and emission reduction an inevitable trend. Currently, prefabricated building has become an important means to achieve the transformation, upgrading, and sustainable development of the construction industry.

Prefabricated building is a production method in which components are prefabricated in the factory and transported to the construction site for assembly. It is characterized by standardized design, component prefabrication, and mechanized assembly construction, aiming to achieve low energy consumption, high quality, and maximize the lifecycle value of the product^6^. With the increasing scale of prefabricated building and the continuous improvement of prefabrication rate, the construction process of prefabricated building involves a large number of different types of prefabricated components, which directly leads to escalating project costs and difficulties in controlling project duration, safety, and other factors according to the planned schedule^7^ For prefabricated building, prefabricated components are important building elements, and the selection of prefabricated component combination schemes has a crucial impact on the overall benefits of prefabricated building. Different types of prefabricated components have varying carbon emissions, and different types of prefabricated components also have different impacts on the overall project cost^8^. The control of construction duration in prefabricated building and the ability to achieve good social, economic, and environmental benefits after project completion are closely related to the selection of prefabricated component combination schemes.

Currently, in the design stage of prefabricated building, the selection of prefabricated components and the screening of combination schemes are often based on single-factor considerations, and the factors taken into account are not comprehensive enough. However, when considering objectives such as cost, construction period, and carbon emissions simultaneously, these objectives are interconnected and mutually constrained. It requires a comprehensive consideration of the balance and mutual influence among them, as well as the handling of multiple and conflicting objectives. This presents a significant challenge in the selection of prefabricated component combinations^9^. If the selection of components can be determined based on the project's requirements and its own context during the conceptual design stage, prefabricated building can maximize cost and time savings while meeting the requirements for prefabrication rate, and minimize carbon emissions in the building. This approach greatly promotes the effectiveness of prefabricated building component selection and the development of prefabricated building. Therefore, the optimization and evaluation research of prefabricated component combination schemes in prefabricated building becomes crucial.

**Figure 1:**
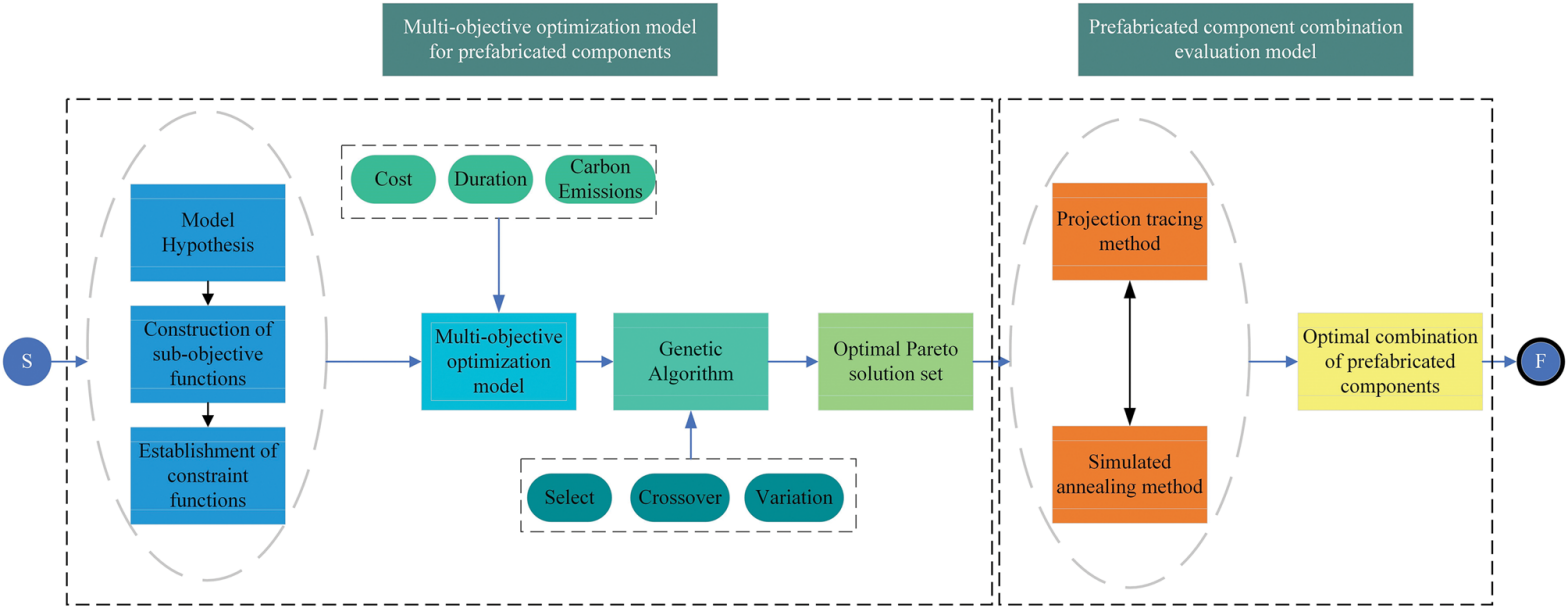
Research framework.

We propose a multi-objective optimization and evaluation method for prefabricated component combination schemes in prefabricated building, considering cost, duration, and carbon emissions. In this method, cost, duration, and carbon emissions are treated as objective functions, and the casting and prefabricated construction processes of components are treated as variables. The prefabrication rate and quality optimization rate are used as constraints. The NSGA-II algorithm is employed to solve the model, resulting in a set of optimal Pareto solutions for prefabricated component combinations. Based on the optimal Pareto solution set, a multi-objective evaluation model is established using simulated annealing optimization projection pursuit method. An optimal prefabricated component combination scheme is selected based on the projection eigenvalues of the scheme. The research approach is illustrated in Fig. 1. This study provides theoretical support and decision-making basis for the scientific and rational selection of prefabricated component combination schemes. Stakeholders can achieve significant overall benefits in prefabricated building by choosing suitable prefabricated component combination schemes for their engineering projects. This also lays the foundation for the scientific and sustainable development of prefabricated building.

## Literature review

### Optimization objectives

In the traditional field of construction engineering, the optimization objectives mainly focus on the traditional project management goals of cost, construction period, and quality. Siemens N used network planning to draw and study the cost-time curve in order to shorten the construction period of projects^10^. Kapur K conducted research on the relationship between cost and construction period and found that they follow a nonlinear quadratic function relationship^11^. Wang et al. conducted multi-objective optimization of cost, construction period, and quality in construction projects, determining the trade-off points among the objectives^12^. Ke constructed different cost-time-quality optimization models based on the differences in decision-makers' management goals and requirements^13^. As research progresses, some scholars also consider incorporating other indicators into the scope of consideration. Talaei Maryam studied the energy consumption of buildings by considering daylighting levels and energy usage intensity as multi-objective optimization objectives^14^. Malik et al. conducted multi-objective optimization of building energy consumption and comfort management^15^. Peng et al. quantitatively analyzed prefabricated building from a sustainability perspective by introducing sustainability objectives into the traditional issues of construction period and cost^16^. Milat et al., considering the complexity of construction projects, managed the impact of uncertainty by treating stability as the objective of multi-objective optimization^17^.

In recent years, the rapid development of industrialized construction has posed severe challenges and threats to global ecological resources and the natural environment, leading to an unprecedented level of importance for sustainable development^18^. The construction industry has long been criticized for its high energy consumption and low efficiency. It not only consumes a large amount of energy but also generates a significant amount of carbon emissions during the construction process, exacerbating the greenhouse effect^19^. In order to effectively reduce carbon emissions, in-depth research on the carbon emissions of prefabricated building can provide valuable guidance for the construction process of prefabricated buildings. Zhou et al. compared the carbon emissions differences between prefabricated building and cast-in-place construction through five stages of construction^20^. Zhao et al. developed a carbon emission energy value factor (Em-CEF) accounting model by combining carbon emission factors and energy value analysis. They conducted a comparative analysis of carbon emissions during the construction phases of traditional, prefabricated, and green material buildings^21^. Mo et al. calculated the carbon emissions of an office building in Hangzhou, Zhejiang, based on comprehensive records and factory data analysis. The results indicated that adopting the PEC structural system can effectively reduce carbon emissions^22^. In terms of optimizing building carbon emissions, different scholars have different research perspectives. Li et al. focused on the window design of building facades and discussed the optimal window-to-wall ratio to achieve the best carbon emission performance^23^. Chen et al. optimized the longitudinal steel section dimensions and areas of T-beams with carbon emissions as the objective, obtaining the optimal combination of rebar diameters^24^. Zhang et al. analyzed the impact of various thermal properties of building enclosure structures, including external walls, roofs, and windows, on building energy consumption and carbon emissions^25^.

Domestic and foreign scholars have conducted a considerable amount of research on building carbon emissions and carbon emission optimization issues from both theoretical and practical perspectives, and have achieved certain research results. Carbon emissions, like project costs, construction period, and quality management, are all manifestations of the comprehensive value of engineering projects. However, the field of construction is complex, involving multiple factors. In order to achieve the goal of the full life cycle carbon emissions of buildings, various technological means and emission reduction measures need to be adopted, which may have an impact on other objectives. Among them, the relationship between carbon emissions and costs is a hot research topic. Guo et al. used an improved optimization algorithm to study that the cost factor will affect the choice of prefabricated assembly technology to influence carbon emissions. Companies can achieve better emission reduction effects by appropriately increasing costs^26^. Langston et al. found a strong linear relationship between embodied carbon emissions and costs by investigating the embodied carbon footprint of new construction and renovation projects^27^. On the other hand, in order to meet the needs of sustainable development, carbon trading has attracted much attention in recent years as an effective policy for controlling greenhouse gas emissions^28^. Carbon trading is an economic tool and policy measure that has developed in response to climate change and reducing greenhouse gas emissions. It refers to the buying and selling of carbon emission rights and trading activities through market mechanisms^29^. Therefore, the implementation of carbon trading policies makes carbon emissions part of a company's business activities, transforming carbon emissions from an external environmental cost to an internal cost for companies. The full lifecycle cost of buildings will be influenced by the amount of carbon emissions^30^. In the construction process, prefabricated buildings, as an important means to alleviate pollution and reduce carbon emissions, can greatly shorten the construction period compared to traditional cast-in-place buildings^31^. However, choosing environmentally friendly materials to reduce the carbon emissions of buildings may require more time for procurement and transportation^32^. Implementing energy-saving and emission reduction technologies and the installation of renewable energy equipment^33^ also require more time, as well as developing emission reduction optimization design schemes^34^ which need additional time for design and verification, all of which can extend the construction period and affect the schedule. At the same time, reducing building carbon emissions typically requires the use of higher quality materials and technologies, indicating a positive correlation between building carbon emissions and quality objectives. Therefore, there is a certain relationship between building carbon emissions and cost, construction period, and quality objectives. It is necessary to consider various factors comprehensively, find the optimal balance point, and reduce building carbon emissions while ensuring the achievement of cost, construction period, and quality objectives.

In summary, research on building optimization started relatively late but has been developing rapidly. Currently, multi-objective optimization studies for buildings should not be limited to traditional objectives such as cost and construction period. More factors need to be considered to improve the overall benefits of buildings. In the development of prefabricated building, carbon emissions have gradually become a key factor that needs to be considered. Currently, research on carbon emissions mainly focuses on carbon accounting and comparative studies, while research on optimizing building carbon emissions is not as in-depth. In the context of industrialized construction, prefabricated components, as composite materials, contain a significant amount of building carbon emissions. However, optimization research focusing on prefabricated components as the optimization object has not received much attention. At the same time, the objectives of cost, construction period, and carbon emissions in building construction are interconnected and mutually constraining. This means that when selecting prefabricated component combinations, it is not possible to consider one factor in isolation; rather, it is necessary to comprehensively consider the balance and interplay among them. Additionally, handling more than one conflicting objective poses a significant obstacle to the selection of prefabricated component combinations.

### Optimization objects

As the basic components of prefabricated building, the selection of prefabricated component combination schemes has a crucial impact on the overall benefits of prefabricated building. Du et al. conducted research on the decision-making of prefabricated component supply chains based on multi-agent theory and proposed a decision support framework for the supply chain^35^. Tao et al. utilized waste generated from prefabricated component production as fuel for recycling, which is beneficial for conserving natural resources and protecting the environment^36^. Du et al. effectively tracked the supply chain of prefabricated components using RFID technology and calculated and studied the costs and benefits at each stage^37^. Loss et al. studied a new type of steel-wood composite floor for multi-story residential buildings, which can be produced in advance as prefabricated modular components and assembled on-site^38^. As research on building carbon emissions deepens, Mao et al. suggest that the embodied carbon emissions of building materials account for 85% of the total lifecycle carbon emissions of buildings^39^. Ji et al. also argue that embodied carbon emissions from building materials are the main source of carbon emissions in both prefabricated and traditional construction^40^. This indicates that a significant portion of carbon emissions during the construction phase comes from the embodied carbon emissions of prefabricated components. Environmental issues add additional complexity to the selection of prefabricated component combinations for prefabricated buildings. Prefabricated components, as the basic components of prefabricated buildings, can have different impacts on the overall project cost depending on the type of prefabricated component used. The construction period and carbon emissions associated with different types of prefabricated components also vary. The rational selection of prefabricated component combinations has a decisive impact on the overall efficiency of prefabricated buildings. Therefore, scholars have also conducted research on the selection of prefabricated component combinations. Chen et al. selected the optimal solution from eight scenarios based on prefabricated rate, rationality of combination schemes, cost, quality optimization contribution, and carbon footprint using a projection tracing dynamic clustering model^41^. Wang et al. constructed an evaluation index system based on prefabricated rate, cost, construction period, and carbon footprint, and then used a fuzzy grey correlation projection method to evaluate and select the best solution from six scenarios^8^. Gao et al. have developed a multi-objective optimization algorithm for prefabricated component combinations to minimize project costs and construction periods to the greatest extent^42^. This indicates that current research on the optimization of prefabricated component combinations involves analyzing predetermined component combinations through enumeration and permutation methods, which is feasible when the number of component types is limited. However, as engineering projects become increasingly complex and the variety and quantity of prefabricated components continue to increase, the existing research has significant limitations. Therefore, the key focus of current research is how to find the optimal prefabricated component solution in an unbounded or non-predefined finite solution space.

The advantages of prefabricated building, such as improved construction quality and shortened construction period, are often difficult to guarantee in actual construction processes. This is mainly due to the influence of different resource constraints and uncertainties during the construction of buildings^6^. In the construction phase of prefabricated building, there is a need for extensive and diverse coordination in the secondary design of prefabricated components, as well as multi-party collaborative construction involving more stakeholders. As a result, the difficulty of construction management increases significantly, and complex environmental constraints need to be addressed. As an important component of the prefabricated building standard system, the evaluation criteria for prefabricated building play a crucial role in guiding the development of prefabricated building. The evaluation criteria for prefabricated building set a minimum requirement for the prefabrication rate of prefabricated buildings. Therefore, the combination of prefabricated components in prefabricated building must meet the minimum standard for prefabrication rate. Compared to traditional cast-in-place construction, prefabricated concrete construction has significant differences in construction techniques and processes. Although there are still some safety and quality issues with prefabricated concrete structures due to the immaturity of technology, the advantages of prefabricated building in terms of quality and building functionality have been proven. The use of different types of prefabricated components has different impacts on the quality of prefabricated concrete construction^43,44^. In order to ensure good quality of buildings, there are also limitations on the use of different types of prefabricated components. Additionally, each construction project has cost control and target construction period, as well as carbon emission restrictions imposed by national carbon quota mechanisms. These constraints make the combination of prefabricated components more complex and challenging.

In conclusion, while prefabricated components are essential elements of prefabricated building, current research in this area mainly focuses on construction quality, supply chain, and cost aspects, with limited studies on the selection and evaluation of prefabricated component combination schemes. Furthermore, the selection of prefabricated component solutions is based on predetermined and limited scenarios for comparison and optimization. This approach has significant limitations when dealing with large and complex real-world scenarios. Existing research is unable to truly improve and promote the widespread application of industrialized construction from the perspective of prefabricated components. Moreover, although prefabricated components as new building materials contain a significant amount of carbon emissions, optimization of carbon emissions focusing on prefabricated component combinations has not received much attention, and there is a lack of multi-objective optimization research in this area.

### Optimization methods

As construction projects become increasingly complex, traditional multi-objective optimization methods are no longer sufficient to solve large-scale and complex problems. Intelligent algorithms have demonstrated good performance in solving engineering optimization problems. Among the intelligent algorithms, widely used ones include Genetic Algorithm (GA), Particle Swarm Optimization (PSO), and Ant Colony Optimization (ACO). Genetic Algorithm has strong search capabilities in the solution space. In the context of prefabricated component combination schemes, where multiple objectives such as cost, construction period, and carbon emissions need to be considered, Genetic Algorithm can effectively explore and maintain multiple valid solutions in the solution space. The objectives of cost, construction period, and carbon emissions in prefabricated component combination schemes are interrelated and mutually constrained. Genetic Algorithm can maintain high diversity in multi-objective optimization problems, while Particle Swarm Optimization and Ant Colony Optimization tend to converge to local optima and struggle to maintain diversity. Genetic Algorithm (GA) exhibits good stability and convergence in multi-objective optimization problems. On the other hand, Particle Swarm Optimization and Ant Colony Optimization have relatively poorer stability and convergence in multi-objective optimization problems. Therefore, Genetic Algorithm is more suitable for optimizing prefabricated component combinations. Genetic Algorithm was proposed by Holland in 1975 and is a stochastic search and optimization algorithm based on the basic principles of Darwin's theory of evolution and Mendel's genetics. Yao et al. applied Genetic Algorithm to optimize the layout of prefabricated building sites, improving the quality of the optimal solutions and reducing the solution time^45^. Song et al. conducted research on the collaborative scheduling of multiple projects in prefabricated building using an improved Genetic Algorithm, providing reference for project managers to develop strategies in uncertain environments^46^. Qu et al. optimized the energy usage intensity, thermal discomfort rate, and dissatisfaction with daylight illuminance of buildings using both multi-objective genetic algorithm and Non-dominated Sorting Genetic Algorithm (NSGA-II)^47^. NSGA-II with an elitist strategy is a fast non-dominated multi-objective optimization algorithm based on Pareto optimal solutions. The basic idea of NSGA-II is inspired by the survival of the fittest principle in nature, where individuals with better fitness survive and evolve, and genes of individuals with good environmental adaptability are continuously passed on to the next generation of individuals^48^. Compared to genetic algorithms, NSGA-II reduces computational complexity, achieves faster computation speed, and provides higher optimization accuracy. Therefore, this study adopts NSGA-II to solve the prefabricated component combination problem.

The results of multi-objective optimization often yield not just a single optimal solution but a set of optimal solutions, representing the optimal trade-offs between different objectives on the Pareto front^45^. When evaluating the prefabricated component combination in prefabricated building, commonly used evaluation methods include Analytic Hierarchy Process (AHP), Fuzzy Evaluation Method, Grey Relational Analysis, and Material Element Analysis. Fan et al. used AHP to assess risks in prefabricated building and scheduling, analyzing the transmission mechanism of risks^49^. Zhang et al. used AHP to identify key factors affecting the quality of prefabricated building and analyze their interrelationships^50^. Cao et al. obtained the optimal ATC model using Grey Relational Analysis to guide building design^51^, while Zhang et al. used an improved Grey Relational Analysis method to determine the weights of hierarchical indicators and constructed a comprehensive evaluation model based on lean knowledge acquisition, integration, and application^52^. To avoid stakeholders making decisions without a clear understanding of how specific decisions will impact future project performance and benefits, the decision-making process is often guided by the intuition and experience of engineers^53^. However, these evaluation methods are subjective, and different weightings can lead to different evaluation results. The setting of weights is often based on the preferences of the evaluators. Projection Pursuit (PP) method, as an objective evaluation method that does not require the setting of weights, is widely used in multi-factor evaluation and optimization^54^. Zhao et al. established and evaluated a dynamic risk assessment model for flood disasters using the Projection Pursuit method. The research results showed that the Projection Pursuit model is reasonable, easy to operate, and has theoretical and practical feasibility, providing a new approach for flood risk assessment^55^. Xu et al. evaluated the resilience of agricultural land–water resource systems using a Projection Pursuit model based on the Sparrow Search Algorithm. The research showed that the model has good performance in terms of solution speed and optimization ability^56^. When evaluating prefabricated component combination schemes, the simulated annealing optimization projection pursuit model can provide an objective and reasonable evaluation. This method, considering multiple objectives such as cost, construction period, and carbon emissions, transforms the evaluation problem of prefabricated component combination schemes into an optimization problem using the projection pursuit model. By applying the simulated annealing algorithm and setting the search space and constraints, the model can comprehensively explore the solution space and find optimal combination schemes. Therefore, in this study, the simulated annealing optimization projection pursuit method is employed to evaluate prefabricated component schemes.

In summary, when solving the multi-objective optimization model for prefabricated component combination in terms of cost, construction period, and carbon emissions, the NSGA-II algorithm demonstrates clear advantages in terms of search capability in the solution space, maintaining diversity of solutions, and stability and convergence compared to other algorithms. In evaluating the obtained prefabricated component combination schemes, the simulated annealing optimization projection pursuit model, as an objective multi-factor evaluation method that does not require setting weights, can comprehensively search the solution space while considering multiple objectives. Combining the NSGA-II algorithm with the simulated annealing optimization projection pursuit model allows for a more reasonable selection of optimal solutions for prefabricated component schemes.

### Research gaps and main contributions

A literature review indicates that scholars are paying increasing attention to the multi-objective optimization, solution finding, and evaluation of prefabricated buildings. Although there has been some progress in the optimization of construction projects, the research process has encountered the following issues:

1. Optimization objectives: The research on building optimization mainly focuses on traditional cost and duration aspects. In the context of building energy efficiency, research on carbon emissions in buildings primarily focuses on carbon accounting and comparative studies with traditional buildings. There is a lack of quantitative optimization research combining carbon emissions of prefabricated buildings with multi-objective optimization.
2. Optimization objects: Prefabricated components, as the basic elements of modular construction, contain a significant amount of carbon emissions as composite materials in the industrialized mode. Currently, research on prefabricated components mainly focuses on construction quality, supply chain, and cost aspects. Research on carbon emission optimization and multi-objective optimization with prefabricated component combinations as the optimization objects has not received much attention.
3. Optimization methods: When solving multi-objective optimization problems, the methods used mainly rely on single algorithms, which may face issues such as local optima, high dependence on initial solutions, and difficulty in handling problem complexity. There is a lack of combination of multiple optimization algorithms.

Based on the above issues, in order to provide sustainable solutions for construction projects considering comprehensive factors, this study proposes a multi-objective optimization and evaluation method for prefabricated component combinations based on cost, duration, and carbon emissions. Firstly, the cost, duration, and carbon emissions optimization objectives of prefabricated components, as well as constraints on prefabrication rate and quality optimization rate, are quantified to establish a multi-objective optimization model. Secondly, NSGA-II is used to solve the multi-objective optimization model and obtain the Pareto solution set that satisfies the constraints. Finally, the simulated annealing optimization projection tracing model is used to evaluate the obtained prefabricated component combination solutions and select the optimal combination based on the projection eigenvalues. The remaining parts include the following sections: The third section introduces the methodology and establishment of the multi-objective optimization model, which mainly includes the solution methods for multi-objective optimization problems and the evaluation of solutions. The fourth section presents the solution results, analyzing the optimization results of the optimal prefabricated component combinations based on actual cases. Subsequently, there is a discussion section that includes comparative analysis of the results, advantages, limitations, and so on. Finally, the study's conclusions and recommendations are provided.

## Methodology

### NSGA-II model for multi-objective optimization

**Figure 2:**
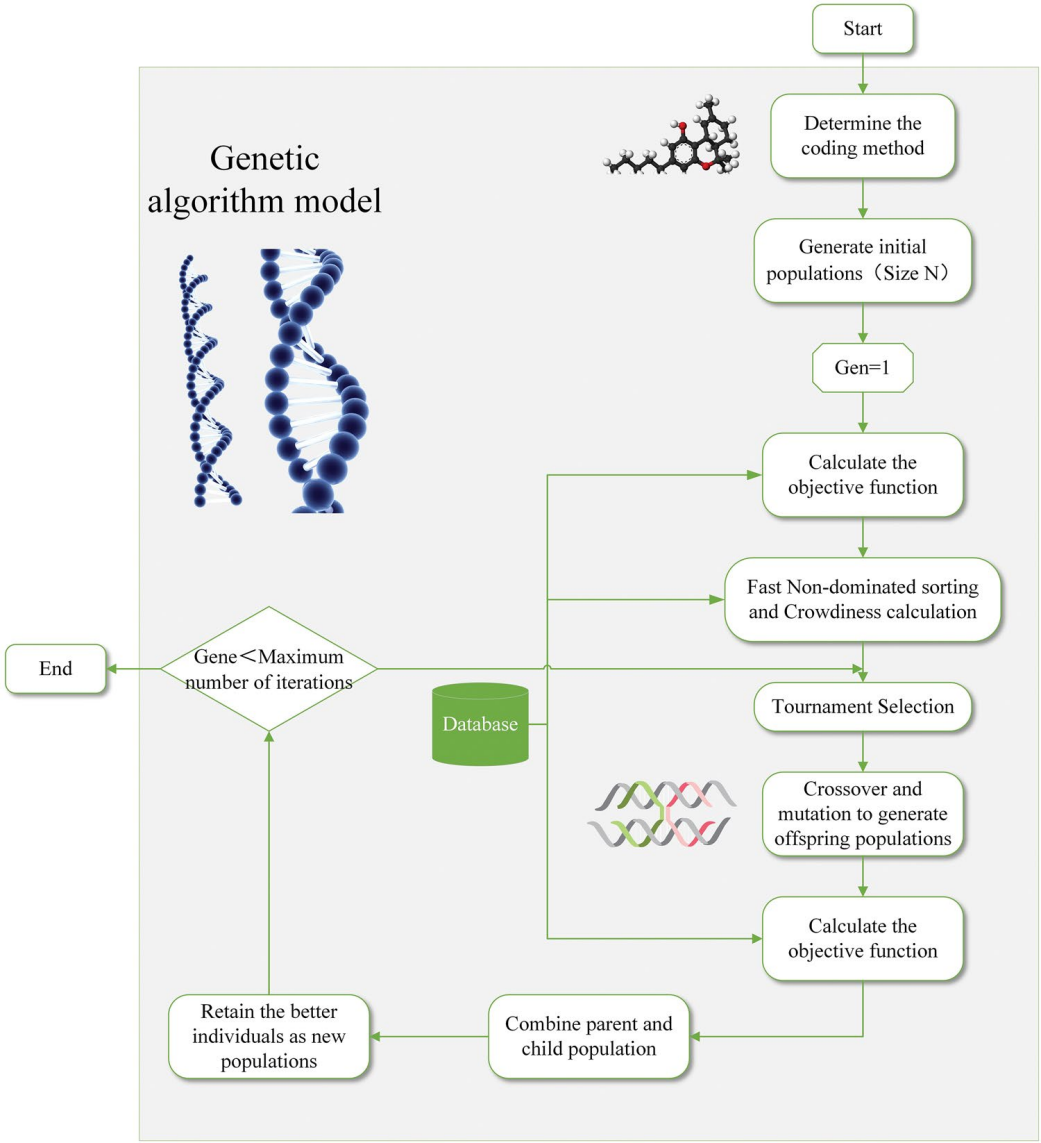
NSGA-II flowchart.

**Figure 3:**
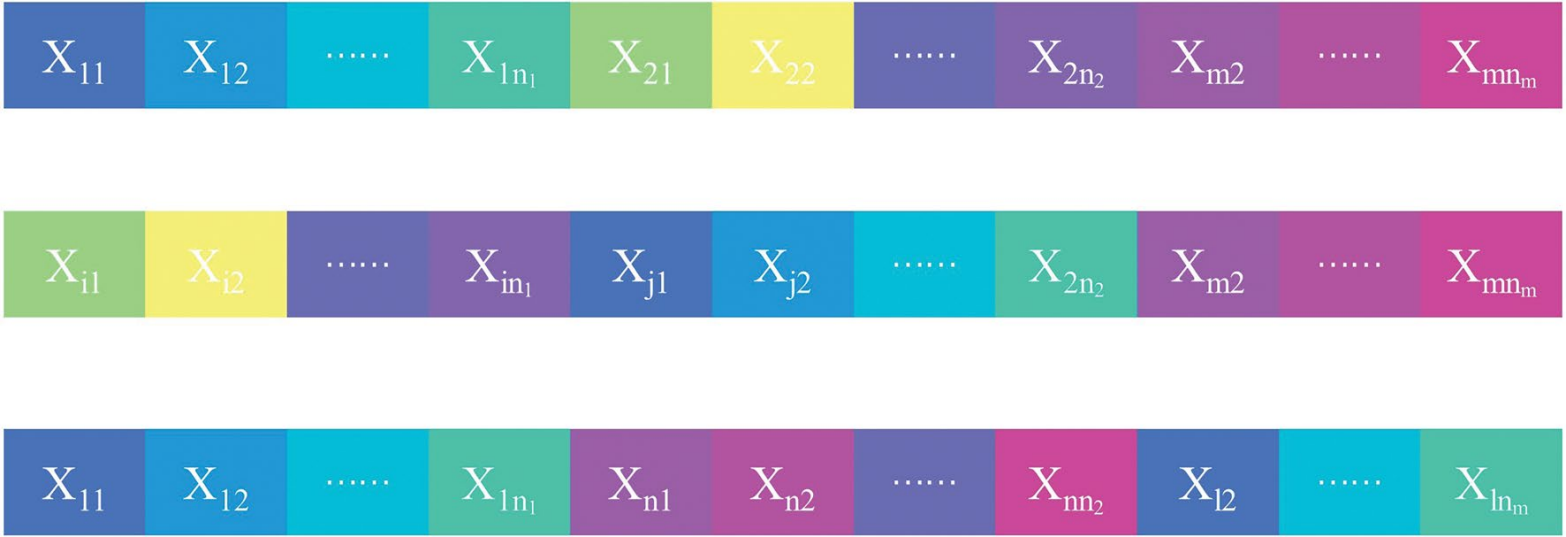
Coding arrangement.

In this paper, the flow of solving the multi-objective optimization model using NSGA-II is shown in Fig. 2. with the following steps:

Step 1: Coding procedure. The arrangement coding approach is used for coding in this paper. The organization coding of chromosomes is depicted in Fig. 3 assuming that each type of component X_i_ will have n_i_ types of construction techniques to select from and that there are m types of various component types on different floors.

Step 2: Make the population start out. The settings of genetic algorithm parameters directly impact its performance, and a reasonable choice and control of these parameters enable the genetic algorithm to search for the optimal solution along the most favorable path^57^. The parameter settings for genetic algorithms are typically determined based on the characteristics of the problem and the search space for solutions^58,59^. These parameters primarily include the initial population size (N), the crossover rate (p_c_), and the mutation rate (p_m_), and these settings directly affect the algorithm's performance and search capabilities^60–62^, as detailed in Table 1.

**Table 1:** The impact of parameter settings on optimization results.

Serial number	Parameter name	Effect on results		Parameter setting
		Larger	Smaller	
1	Population size (N)	Expanding the algorithm's search space coverage can enhance the probability of global search but may also lead to increased computational costs and running time	This results in the algorithm getting trapped in local optima, leading to reduced search capability, but with lower computational costs	Typically, N = 20–200
2	Crossover rate (p_c_)	It can increase the diversity of the population, reducing the likelihood of premature convergence, but it may also result in the population converging prematurely	It will reduce the exchange of information between individuals, leading to a decrease in population diversity and weakening search capability, but it can prevent premature convergence of the population	Typically, p_c_ = 0.6–1.0
3	Mutation rate (p_m_)	It increases the mutation probability of individuals to enhance the diversity of the population and prevent falling into local optima. However, it may impact the convergence speed and stability of the algorithm	Setting a small mutation probability will reduce the mutation rate of individuals, decrease the diversity of the population, which may lead to premature convergence. However, it also helps stabilize the convergence process of the algorithm	Typically, p_m_ = 0.005–0.01

Although the significance of genetic algorithm parameters has been clearly outlined, there is no unified theoretical basis to guide the selection of parameters, and most parameters are obtained through repeated experiments to determine the optimal values^63^. The steps mainly involve determining the population size N, setting initial values for the crossover rate (p_c_) and mutation rate (p_m_) based on the complexity of the research problem to obtain better solutions. Parameters can be fine-tuned within a small range to improve the computational results. Repeat these steps until the most scientific parameters are determined. The number of individuals in the initial population is set as N.

Step 3: Make the first evolutionary generation of the population that is generated Gen = 1, calculate the fitness level of the first N individuals in accordance with the objective function, perform fast non-dominated sorting, congestion calculation, and determine whether the fitness level meets the termination criterion or not. If it does, the generation is terminated; if not, it moves on to Step 4 for execution.

Step 4: The population produced by Step 3 is put through a tournament selection process, which chooses people with high adaptability and weeds out people with low adaptation.

Step 5: Crossover, mutation to create a population of the next generation, objective function calculation for each population member, elite retention strategy, sequential fast non-dominated sorting, congestion calculation to create the population, and the population with the best members as the new population;

Step 6: Determine whether the iteration number Gen is equal to the maximum iteration number. If it is less than the maximum iteration number, then Gen = Gen + 1 and move on to Step 3. Return the execution result as the new generation population to run Step 3. If not, the algorithm stops the operation;

### Cost objective model

In this paper, the cost of components is mainly divided into six parts: labor cost, material cost, machinery cost, management fee, profit and tax, of which the proportion of management fee, profit and tax is selected with reference to the comprehensive quota of the project location. Therefore, the cost accounting for different types of components is shown in the following Eqs. (1), (2).

1 $$ C = \sum\limits_{i = 1}^{n} {C_{i1} X_{i} + C_{i2} (1 - X_{i} )} $$

2 $$ C_{ij} = \sum\limits_{k \in K} {p_{ij}^{k} c_{ij}^{k} + m_{ij}^{k} c_{ij}^{k} + s_{ij}^{k} c_{ij}^{k} + M_{ij} + O_{ij} + T_{ij} } $$

where C is the total cost of the project; n is the total number of types of components; i denotes the i-th component; j denotes the j-th construction techniques, j = 1,2, j = 1 denotes the cast-in-place technique, j = 2 denotes the prefabricated technique; C_ij_ is the cost of the j-th construction techniques of the i-th component; X_i_ = 0 or 1, X_i_ = 0 denotes the prefabricated technique of the i-th component, X_i_ = 1 denotes the cast-in-place technique of the i-th component; $p_{ij}^{k} ,\;m_{ij}^{k} ,\;s_{ij}^{k}$ is the consumption of man, material and machine in the k-th under the j-th construction techniques of the i-th kind of component; $c_{ij}^{k}$ is the unit cost of man, material and machine in the k-th under the j-th construction techniques of the i-th kind of component; M_ij_, O_ij_, T_ij_ are the overhead, profit and tax under the j-th construction techniques of the i-th kind of component, respectively.

### Construction of duration target model

By analyzing the resources that can be allocated to each process of the project, as well as the external environment, policies and other relevant elements of the construction conditions, the construction duration of each process can be determined^64^. In this paper, based on the enterprise time quota of an enterprise in Shenzhen, the duration of the processes included in the construction of the component is calculated, so as to summarize the duration required for the component, which is calculated as shown in Eqs. (3), (4).

3 $$ T = \sum\limits_{i = 1}^{n} {T_{i1} X_{i} + T_{i2} (1 - X_{i} )} $$

4 $$ T_{ij} = \sum\limits_{l \in L} {\frac{{n_{ij}^{l} t_{ij}^{l} }}{{a_{ij}^{l} b_{ij}^{l} }}} $$

where T is the total duration of the project; n is the type of component; i represents the i-th component; j represents the j-th construction techniques, j = 1,2, j = 1 represents the cast-in-place technique, j = 2 represents the prefabricated technique; T_ij_ is the duration of the j-th construction techniques for the i-th component; X_i_ = 0 or 1, X_i_ = 0 indicates that the i-th component adopts the prefabricated technique, and X_i_ = 1 indicates that the i-th component adopts the cast-in-place technique; $n_{ij}^{l}$ is the consumption required for the l-th construction technique under the j-th construction techniques of the i-th component; $t_{ij}^{l}$ is the time quota of the l-th construction techniques under the j-th construction techniques of the i-th component; $a_{ij}^{l}$, $b_{ij}^{l}$ is the number of shifts arranged for the l-th construction technique under the j-th construction techniques of the i-th component and the number of people in each shift.

### Carbon emission target modeling

In this paper, the carbon emission of prefabricated components is calculated mainly based on the “Building Carbon Emission Calculation Standard” (GB/T 51366-2019)^65^, and the basic equation of carbon accounting is: carbon emission of the building = energy and material consumption × Carbon Emission Factor, and the carbon emission of the components should be calculated according to different stages, and the results of the segmented calculation should be accumulated to get the carbon emission of its whole life cycle. The physical stage of the building is mainly divided into three stages: the production of building materials, the transportation of building materials and the construction of the building, so the calculation of the carbon emission of the components is shown in Eqs. (5)–(9):

5 $$ E = \sum\limits_{i = 1}^{n} {E_{ij} X_{i} + E_{ij} (1 - X_{i} )} $$

6 $$ E_{ij} = E_{scij} + E_{ysij} + E_{jzij} $$

7 $$ E_{{{\text{scij}}}} = \sum\limits_{k = 1}^{n} {M_{ijk} F_{ijk} } $$

8 $$ E_{{{\text{ysij}}}} = \sum\limits_{k = 1}^{n} {M_{ijk} D_{ijk} T_{ijk} } $$

9 $$ E_{{{\text{jzij}}}} = \sum\limits_{k = 1}^{n} {N_{ijk} NF_{ijk} } $$

where E is the total carbon emission of the project; n is the type of component; i denotes the i-th component; j denotes the j-th construction techniques, j = 1,2, j = 1 denotes cast-in-place technique, j = 2 denotes prefabrication technique; E_ij_ is the carbon emission under the j-th construction techniques of the i-th component; X_i_ = 0 or 1, X_i_ = 0 denotes that the prefabrication process is adopted in the i-th component, X_i_ = 1 denotes that the cast-in-place technique is adopted in the i-th component. E_sij_, E_ysij_, and E_jzij_ are the carbon emissions of the production, transportation, and construction phases of building materials under the j-th construction techniques of the i-th component, respectively; M_ijk_, F_ijk_, D_ijk_, and T_ijk_ are the consumption of the k-th major building materials under the j-th construction techniques of the i-th component, the carbon emission factor, the average transportation distance, and the carbon emission factor of the transportation distance per unit weight, respectively; N_ijk_, NF_ijk_ are the total consumption of the k-th energy source for the j-th construction techniques of the i-th component and the carbon emission factor of the energy source.

### Constraint construction of precast rate

The case used in this paper is located in Shenzhen, Guangdong Province, so the calculation of precast rate mainly adopts the local standard of Shenzhen. The calculation method of precast rate in the Calculation Rules for Precast Rate and Assembly Rate of Residential Industrialization Projects in Shenzhen issued by Shenzhen is shown in Eq. (10).

10 $$ {\text{V}}_{{\text{Prefabrication rate}}} = \frac{{{\text{V}}_{{\text{Standard floor prefabricated concrete components}}} }}{{{\text{V}}_{{\text{Standard layer full volume}}} }} \times 100\% $$

From the above formula precast rate calculation we can see that the overall precast rate is the sum of the precast rate of each component, so the precast rate of each prefabricated component is the ratio of the volume of the different components to the overall volume of the component, so for the calculation of the precast rate of the project is as shown in Eqs. (11), (12).

11 $$ A = \sum\limits_{i = 1}^{n} {A_{i1} X_{i} + A_{i1} (1 - X_{i} )} $$

12 $$ A_{{{\text{ij}}}} = \frac{{V_{{\text{Prefabricated components}}} }}{{V_{{\text{Total volume}}} }} $$

where A is the total prefabricated rate of the project; n is the type of components; i indicates the i-th component; j indicates the j-th construction techniques, j = 1,2, j = 1 indicates the cast-in-place technique, and j = 2 indicates the prefabricated technique; A_ij_ is the prefabricated rate under the j-th construction techniques for the i-th type of component; X_i_ = 0 or 1, X_i_ = 0 indicates that the i-th type of component adopts prefabricated technique, and X_i_ = 1 indicates that the i-th type of component adopts cast-in-place technique; V_Prefabricated components_ is the volume of the i-th type of component adopting the prefabricated technique; V_total volume_ is the total volume of the component.

### Constraint construction for quality optimization contribution rate

Quality Function Deployment (QFD) is a systematic quality management tool used to translate customer requirements into specific design requirements for products or services, and to convert these design requirements into specific operational guidelines in the actual production process^66,67^. The key to implementing QFD is a waterfall-like decomposition approach, which, through cascading layers, explores the importance of different components in relation to quality optimization compared to traditional construction methods^68^. Additionally, QFD allows for quantifiable assessment, using a quantitative scoring system to evaluate the quality optimization contribution rate of prefabricated components compared to cast-in-place components^69^. This enables the exploration of the degree of quality optimization relative to traditional construction methods under different component combinations and prefabrication rates. Therefore, this paper introduces Quality Function Deployment (QFD) to quantify the quality optimization contribution rate indicator^70^, which can effectively investigate the importance of different components in relation to quality optimization compared to traditional construction methods.

**Figure 4:**
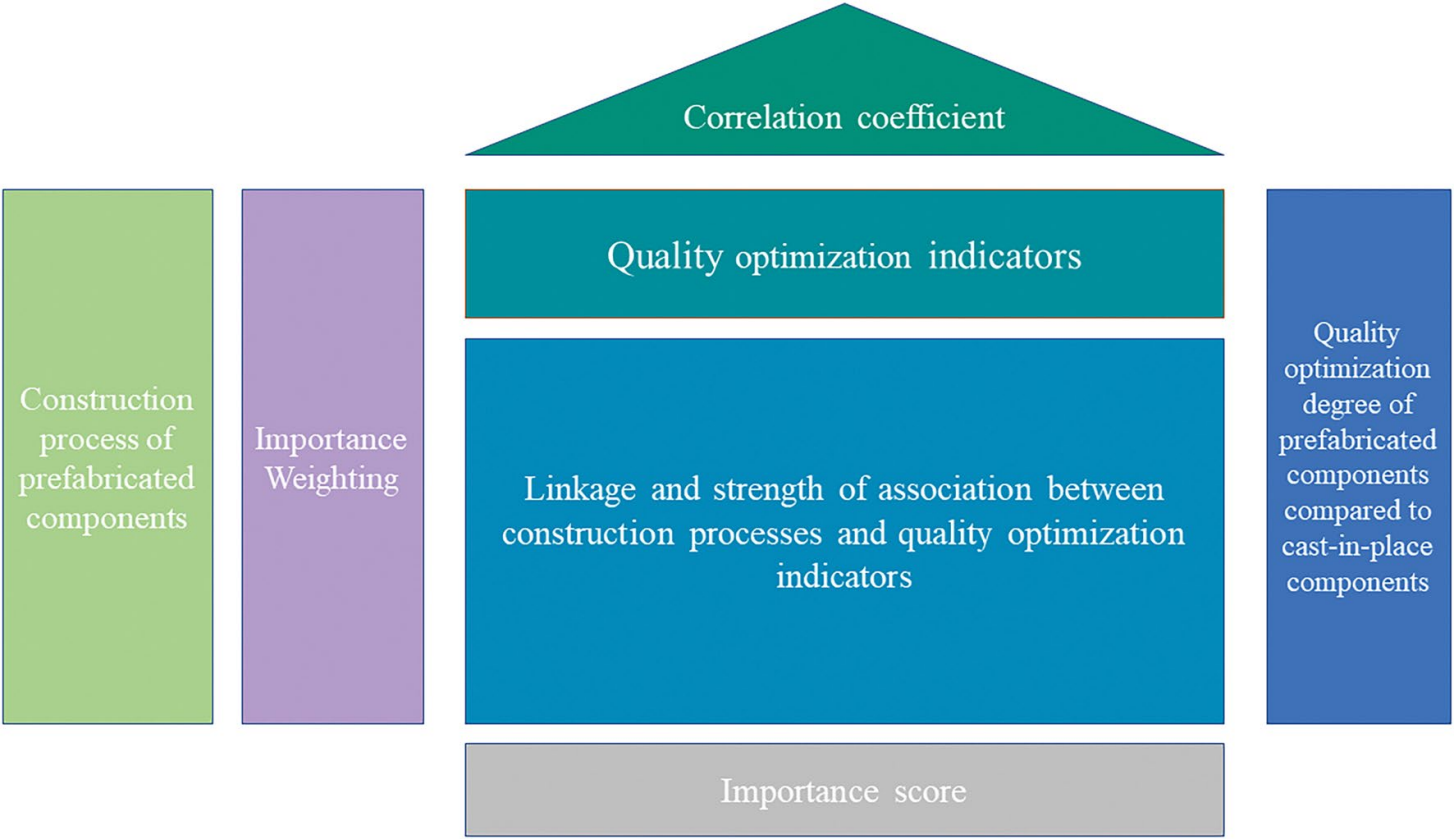
Construction of the first level of the mass house.

The House of Quality is a fundamental tool in QFD, and by establishing the House of Quality, the transformation of each level of elements can be completed, converting the core processes into tangible expressions^71^. The QFD House of Quality consists of two attributes: customer requirements and engineering measures^72^. The first level of the quality house is built as illustrated in Fig. 4. QFD is really the level of correlation between each process and each quality indicator.

As seen in Fig. 4, the quality house is made up of six parts: the left wall, ceiling, room, roof, right wall, and basement. Each part has a unique meaning, which is depicted in the image. Each value Z_nm_ in the quality house represents the degree of connection between the n-th process and the m-th quality index. Z_nm_ is assessed using the expert scoring system, and the score range of 1 to 5 represents how well a process has been optimized for quality in comparison to more conventional building methods. Through the relationship matrix can be calculated for each construction techniques accounted for the proportion of the quality optimization of the whole project, the calculation formula is:

13 $$ v_{{{\text{nm}}}} = \sum\limits_{{{\text{m}} = 1}}^{{\text{m}}} {w_{{\text{m}}} z_{{{\text{nm}}}} } $$

where $w_{{{\text{nm}}}}$ is the weighting factor of each quality indicator.

We use Structural Equation Modeling (SEM) to quantify the weight of quality indicators w_nm_ in prefabricated construction. Prefabricated construction, as a complex field involving multiple factors, is influenced by numerous factors. These factors vary in their sources, timing, and degree of impact, and they intertwine and interact with each other^70^. Structural Equation Modeling (SEM) is a statistical analysis method used to explore complex relationships between variables and predict causal relationships between variables^73^. SEM can consider relationships between multiple variables simultaneously, and the size and direction of path coefficients can reflect the strength and direction of relationships between different variables, visually displaying the importance of different factors and providing a scientific basis for decision-making^74,75^. Therefore, path coefficients have a certain theoretical basis and reliability in determining the importance of factors.

The second layer of the quality home primarily creates a connection between each building process p_n_ and the construction type X_h_. The second layer's quality house matrix is a 0–1 matrix, scoring 1 if a process is used on the precast component and 0 otherwise. It is feasible to summarize the important scores of the various prefabricated components in terms of the value degree S_h_ by building the second layer of the quality house, which is calculated using the following formula:

14 $$ S_{{\text{h}}} = \sum\limits_{{{\text{n}} = 1}}^{n} {{\text{g}}_{hn} v_{hn} } $$

where $v_{hn}$ is the importance weight obtained for the first level of the mass house, h is the number of components, and g_hn_ is the value of each term of the 0–1 matrix for the second level of the mass house.

The quality optimization rate of prefabricated components can be obtained by carrying out the calculation of the proportion of different components through the importance score values of different components, the numerator is the importance score situation of each component, and the denominator is the importance score situation of the component assuming that the optimization degree is all full scores. On this basis, by calculating the proportion of the component quality optimization rate to the sum of the total component quality optimization rate, the quality optimization contribution rate of prefabricated components Q can be obtained, so the quality optimization contribution rate of prefabricated component combination is calculated as follows:

15 $$ Q = \sum\limits_{i = 1}^{n} {Q_{ij} X_{i} + Q_{ij} (1 - X_{i} )} $$

where Q is the quality optimization contribution rate of the project; n is the type of components; i denotes the i-th component; j denotes the j-th construction techniques, j = 1, 2, j = 1 denotes the cast-in-place technique, j = 2 denotes the prefabrication process; Q_ij_ is the quality optimization contribution rate of the i-th component under the j-th construction techniques; X_i_ = 0 or 1, X_i_ = 0 denotes that the i-th component adopts prefabrication process, X_i_ = 1 denotes that the i-th component adopts the cast-in-place technique; m_i_ denotes that there are m_i_ types of construction techniques for the i-th component.

**Figure 5:**
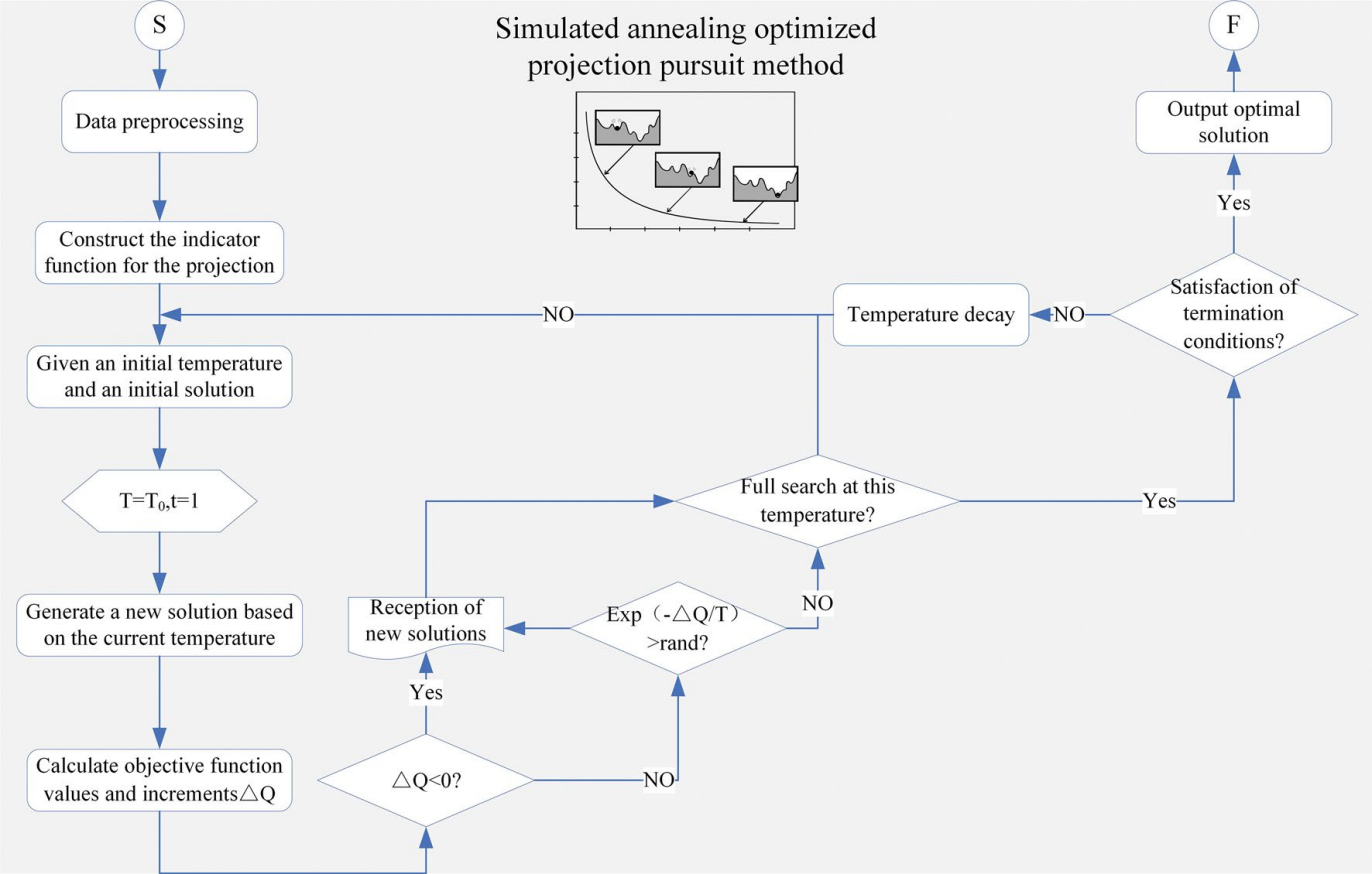
Flowchart of the simulated annealing algorithm to optimize the projection pursuit method.

Simulated annealing algorithm to optimize the projection pursuit evaluation model. In order to get the optimum projection that can accurately reflect the original data, the objective function created by the projection pursuit is optimized in this study using the simulated annealing approach. The projection pursuit model flow chart Fig. 5 is optimized using the simulated annealing approach, and the calculation procedure is as follows:

Step 1: Data preparation. In the prefabricated component combination solution, normalize the cost, time, and carbon emission optimization objectives.

Step 2: Create the projection's index function. In order to convert the multi-objective optimization problem into a one-dimensional projected eigenvalue sequence, convert the cost, duration, and carbon emission high-dimensional data into low-dimensional data. The projection value of sample i in the projection direction is set to the following value, assuming that a_j_ is the projection direction vector:

16 $$ z_{i} = \sum\limits_{j = 1}^{n} {a_{j} x_{ij}^{*} ,\quad i = 1,\;2,\; \ldots ,\;n} $$

In selecting the optimal projection direction, the indicator is considered to be optimal when it is infinitely close to the maximum value, and the projection indicator function Q(a) is expressed as:

17 $$ Q(a) = S(a) \times D(a) $$

18 $$ S(a) = \sqrt {\frac{{\sum\limits_{i = 1}^{n} {\left( {z_{i} - \overline{z}} \right)}^{2} }}{(n - 1)}} $$

19 $$ D(a) = \sum\limits_{i = 1}^{n} {\sum\limits_{k = 1}^{n} {(R - r_{ik} )f(R - r_{ik} )} } $$

20 $$ r_{ik} = \left| {z_{i} - z_{k} } \right| $$

where S(a) is the inter-class dispersion, D(a) is the local density of the projected values, is the mean value of the projection, R is the window radius of the local density, and the function f is the unit step function, which takes the value of 1 when R ≥ r_ik_, and 0 otherwise.

Step 3: Generate the prefabricated component combination solution randomly, and regard it as the optimal solution to calculate the objective function.

Step 4: Set the initial temperature, and set the initial number of iterations: t = 1;

Step 5: Randomly vary the current optimal prefabricated component solution and generate a new prefabricated component solution and calculate its objective function value and increment $\Delta Q$;

Step 6: Accept the solution as the current optimal best solution when $\Delta Q$ < 0; when $\Delta Q$ ≥ 0, select the new solution as the optimal solution through $P = \exp \left( { - \frac{\Delta Q}{T}} \right)$;

Step 7: If t< maximum iteration number, then t = t + 1, return to Step 3:

Step 8: When there is no complete cooling, ${\text{T = }}T\left( {\text{t}} \right)$,enter the process Step 2; when for the cooling state, directly output the current results.

Step 9: According to the best projection a*, in accordance with Eq. (21), calculate the best projection value of each prefabricated component solution $z_{i}^{*}$, sorted by the value of $z_{i}^{*}$ from the largest to the smallest.

21 $$ z(i) = \sum\limits_{j = 1}^{p} {a(j)x(i,\;j),\quad i = 1,\;2,\; \ldots ,\;n} $$

## Results

### Project background

The chosen project is a frame building with 11 floors above ground that is part of the Harmony Garden Project in Shenzhen, Guangdong Province. Two construction techniques, cast-in-place and prefabrication, are selected, and PKPM-PC 2023 R 2.4 structural calculation and analysis software (https://product.pkpm.cn/downloadlist) and Quanta BIM civil metrology platform GTJ2021 (Version number: 1.0.29.2) (https://www.glodon.com/product/145.html) are applied to model the building respectively, and the model assumptions are as follows:

1. The study focuses primarily on the combination of columns, beams, walls, boards, stairs, so the total cost of the building is the sum of the costs of these components. Foundation costs, door and window costs, and other component costs are not included in the total cost, and the duration of the duration and carbon emissions are similar to this.
2. No thought is given to how the choice of cast-in-place or prefabricated building techniques for the components and their impact on the structure of other components may modify the structural stresses of the building structure.

**Figure 6:**
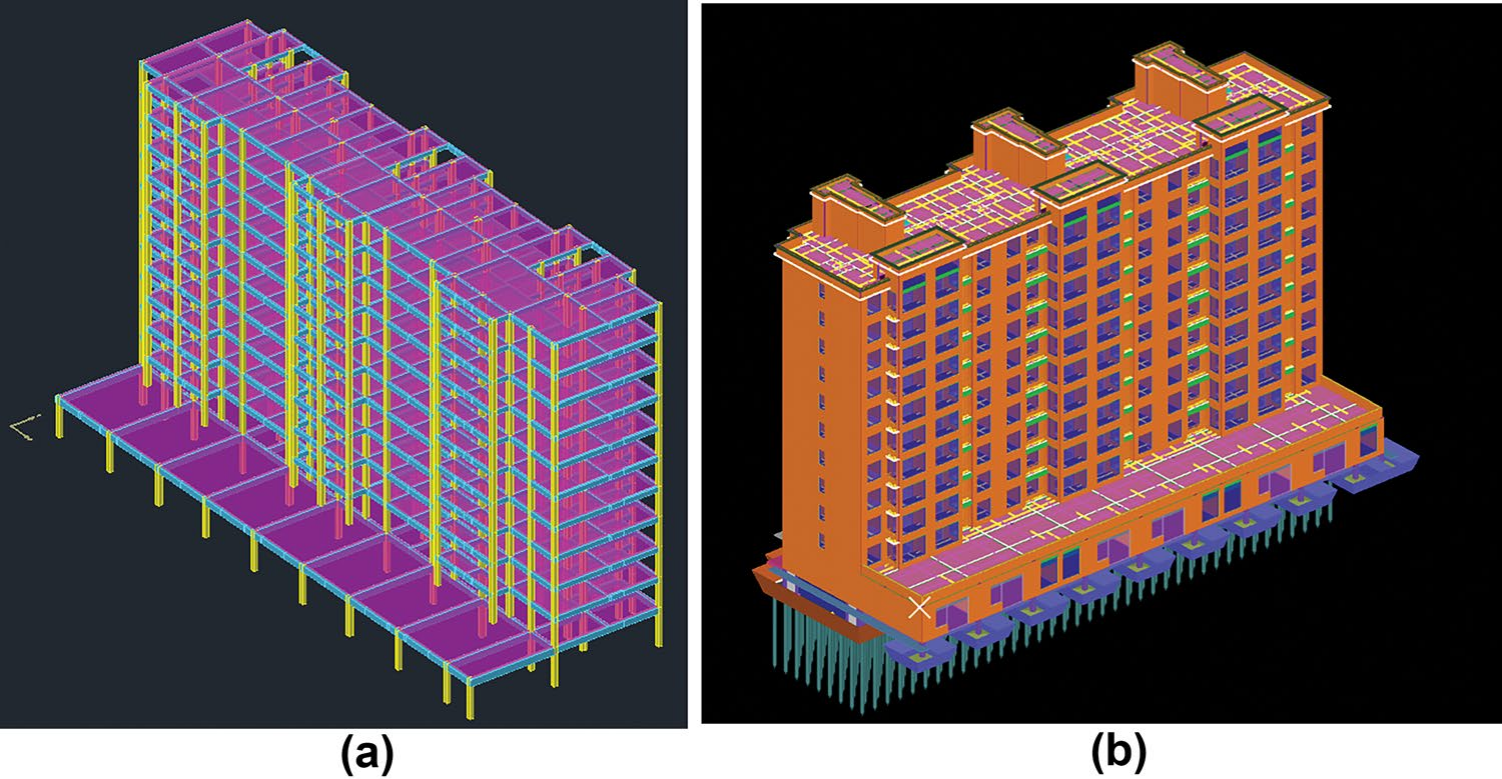
3D model of the building.

The three-dimensional building model created using PKPM-PC 2023 R 2.4 structural calculation and analysis software is shown in Fig. 6a, and the three-dimensional model created using Quanta's BIM civil construction measuring platform, GTJ2021 (Version number: 1.0.29.2), is shown in Fig. 6b. The building structure construction drawings are created using the PKPM-PC 2023 R 2.4 structural calculation and analysis software, and then imported into GTJ2021 (Version number: 1.0.29.2), Quanta's BIM civil construction measurement platform, to obtain the engineering quantity of each component. Equations (1)–(12) are used to compute the cost, duration, carbon emission, and precast rate of each component under the cast-in-place and prefabricated building construction techniques, respectively. The results are displayed in Table 2. In the table i = 2, 3, 4, … 10.

**Table 2:** Cost, duration, carbon emissions, precast rate for each component.

Floor	Cast-in-place component						Prefabricated component			
	Number	Type	Cost (yuan) × 10^4^	Duration (day)	Carbon emissions (t)	Precast rate (%)	Cost (yuan) × 10^4^	Duration (day)	Carbon emissions (t)	Precast rate (%)
First floor	1	Columns	10.95	12	62.48	0	13.41	6	54.87	0.82
	2	Beam	15.97	14	53.47	0	19.97	8	46.75	1.90
	3	Wall	29.31	21	121.41	0	34.04	11	108.65	8.41
	4	Boards	15.57	18	64.48	0	18.60	10	56.08	1.74
	5	Stairs	1.75	7	4.45	0	1.96	3	3.33	0.17
Second floor	6	Columns	4.77	9	25.92	0	5.73	4	20.88	0.44
	7	Beam	7.99	11	27.93	0	8.47	6	19.83	0.81
	8	Wall	20.05	18	79.66	0	22.31	10	69.09	5.52
	9	Boards	8.25	16	31.78	0	9.32	8	26.98	0.87
	10	Stairs	1.67	7	4.10	0	1.80	3	3.06	0.15
Third to eleventh floors	5i + 1	Columns	4.51	9	24.25	0	5.69	4	19.10	0.42
	5i + 2	Beam	7.99	11	27.95	0	8.49	6	19.89	0.80
	5i + 3	Wall	20.00	16	79.34	0	22.37	10	69.26	5.51
	5i + 4	Boards	8.25	14	31.78	0	9.32	8	26.98	0.87
	5i + 5	Stairs	1.67	7	4.10	0	1.80	3	3.06	0.15

Table 2 shows the summary of the cost, duration, carbon emission and precast rate of each component, for example, the cost of the cast-in-place column component of the first floor is 10.95 × 10^4^, the duration is 12 days, the carbon emission is 62.48 t, and the precast rate is 0%, and the other components are similar. From Table 1, it can be seen that: there are differences in the cost, duration and carbon emission of different components, and all the three floors and above are standard floors, so the cost, duration, carbon emission, and precast rate are the same for all the components of each floor. Under both cast-in-place and prefabricated building techniques, the values of cost, duration, and carbon emission of each component differ greatly and have influence on each other, so it is not possible to find an optimal prefabricated component combination solution to optimize cost, duration, and carbon emission, but only to find a relatively optimal combination solution. In order to solve the multi-objective optimization model using NSGA-II, each component is numbered, as shown in Table 2.

**Figure 7:**
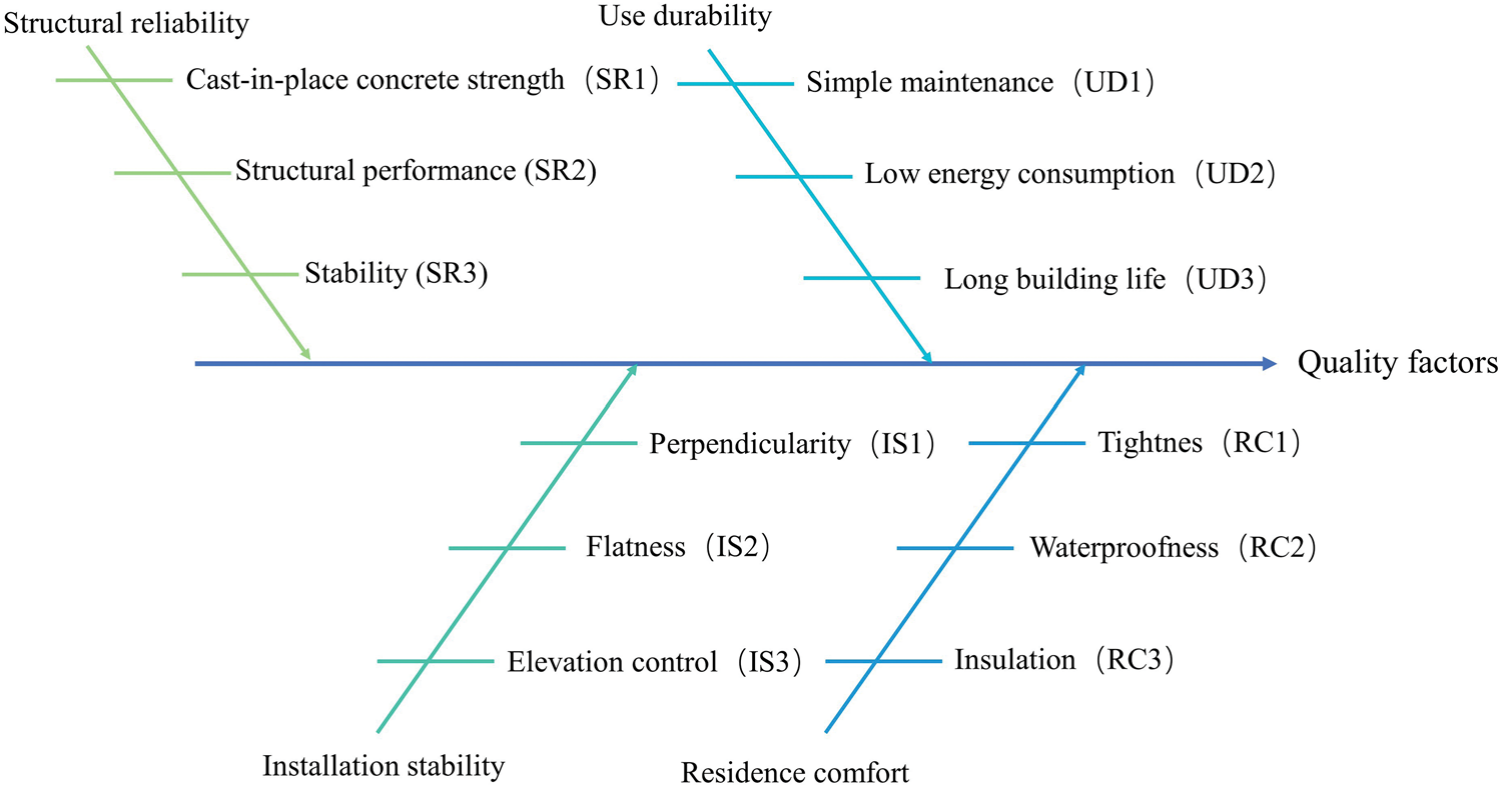
Quality influencing factors.

By reviewing relevant literature from domestic and international sources, this paper conducts an in-depth analysis of the construction process of prefabricated buildings in practice. It compares the cast-in-place and prefabricated construction processes, and after careful investigation and screening, constructs 12 detailed influencing factors of prefabricated building quality from the dimensions of structural reliability^76^, use durability^77^, installation stability^78^, and residential comfort^79^. These factors are illustrated in Fig. 7, and a detailed analysis can be found in Wang, Xu, et al.^70^.

The four major categories of the quality-influencing factors—Residential comfort (RC), Use durability (US), Installation stability (IS), and Structural reliability (SR)—as well as the three sub-categories that each major category contains—are shown in Fig. 7.; they are denoted by 1, 2, and 3, respectively. Using the quality function development (QFD) method, the contribution of prefabricated components to quality optimization was measured. To determine the significance of prefabricated components, a questionnaire was sent to the relevant stakeholders. A five-point Likert scale with a scale from “1” to “5” was used to rate the degree of influence. A total of 221 questionnaires were distributed online; of those, 197 were legitimate, making the percentage of valid surveys at 89.14%. The reliability of the questionnaire was examined using SPSS v22.0, and the results showed that the data had a high degree of reliability and passed the reliability test. The Cronbach's coefficient of this questionnaire was 0.779 > 0.6, and the CITC values were all higher than 0.5. Using the KMO measure and the Bartlett's sphere test, the validity of the latent and observable variables in the model of quality affecting factors was examined. The scale's KMO value was 0.754 > 0.5 and the Bartlett's sphericity test result was 0.000 < 0.05, which made the scale valid and suited for factor analysis. The importance of quality influencing factors is calculated using Structural Equation Modeling (SEM). The fit of the structural equation model is determined by whether the fitness indices are within an acceptable range. Based on current research on SEM structural equation models, Table 3 presents the model fit indices and their acceptable ranges.

**Table 3:** Selection of model fitness indicators and acceptable ranges.

Serial number	Indicator name	Acceptable range	References
1	χ^2^/df	≤ 3.00 Good fit	^80,81^
2	GFI	> 0.80	^80,82^
3	AGFI	> 0.80	^80,81^
4	CFI	> 0.90 Good fit	^80^
5	RMSEA	< 0.05 Good fit	^80^
		< 0.08 Better fit	
		< 0.10 General fit	

**Figure 8:**
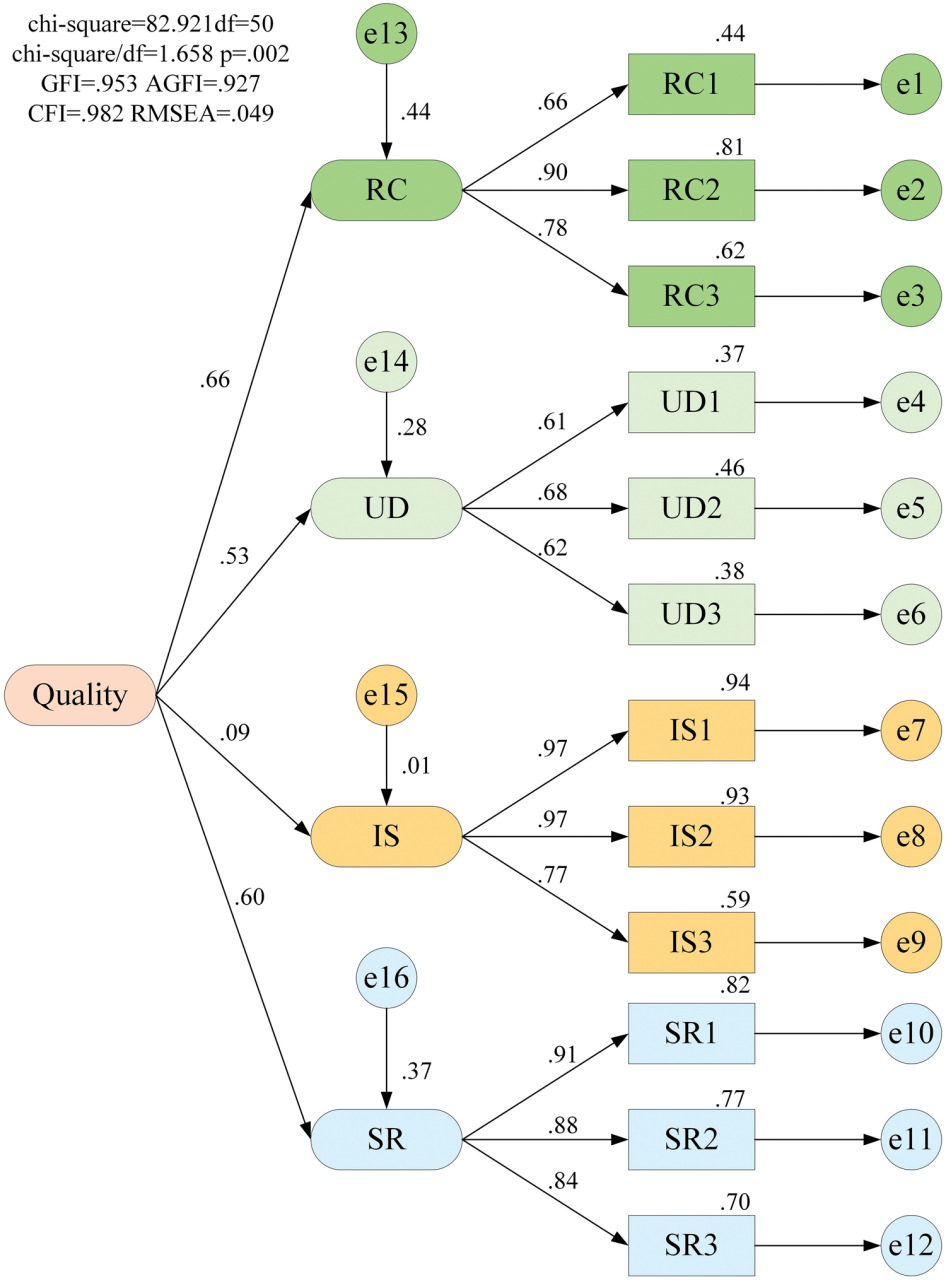
Structural equation modelling of quality influencing factors.

The results of the analysis of the survey data using IBM SPSS AMOS Version 24 software (http://www.spss.com.hk/amos/) to create the structural equation model of quality factors are displayed in Fig. 8.

The structural equation model of quality-influencing factors is depicted in Fig. 8. The path coefficients between the elements, which indicate the importance of the factors, are represented by the numbers in the model. Consider living comfort as an example. The path coefficient of the living comfort index for quality is 0.66. The path coefficients of RC1, RC2, and RC3 for RC are 0.66, 0.90, and 0.78, respectively. The structural equation modeling fitness metrics constructed in this paper are shown in the following Table 4.

**Table 4:** Judgment scale for fitness indicators.

Serial number	Indicator name	Fitting Indicator Values	Acceptable range	Judgment results
1	χ^2^/df	1.658	≤ 3.00	Good fit
2	GFI	0.953	> 0.80	Good fit
3	AGFI	0.927	> 0.80	Good fit
4	CFI	0.982	> 0.90	Good fit
5	RMSEA	0.049	< 0.05	Good fit

The Table 4 shows that all fit indices of the structural equation model for quality influencing factors meet the requirements, indicating that the structural equation model has a good fit. Therefore, the importance of quality influencing factors is quantitatively analyzed through the path coefficients of each indicator, following the steps below:

1. Let *A*_*i*_ (*i* = 1, 2, 3, 4) be the value of the contribution of the first-level indicator to the object of study, hereinafter referred to as weight 1, and let *B*_*i*_ be the path coefficient between the object of study and the first-level indicator, then the formula for calculating weight 1 is shown in formula (22).
  22 $$ A_{i} = \frac{{B_{i} }}{{\sum\limits_{i = 1}^{4} {B_{i} } }} $$
2. Let $C_{i,j}$ be the contribution value of each second-level indicator to its first-level indicator, hereinafter referred to as weight 2, and let $b_{i,j} \;(j = 1,\;2,\; \ldots ,\;k)$ be the path coefficient between the second-level indicator and the first-level indicator, then the formula for calculating weight 2 is shown in Eq. (23).
  23 $$ C_{i,j} = \frac{{b_{i,j} }}{{\sum\nolimits_{j = 1}^{k} {b_{i,j} } }} $$
3. Let the contribution value of secondary indicators to the research object be A_j_, hereinafter referred to as the total weight, which is calculated as shown in formula (24).
  24 $$ A_{j} = A_{i} \times C_{i,j} $$

**Figure 9:**
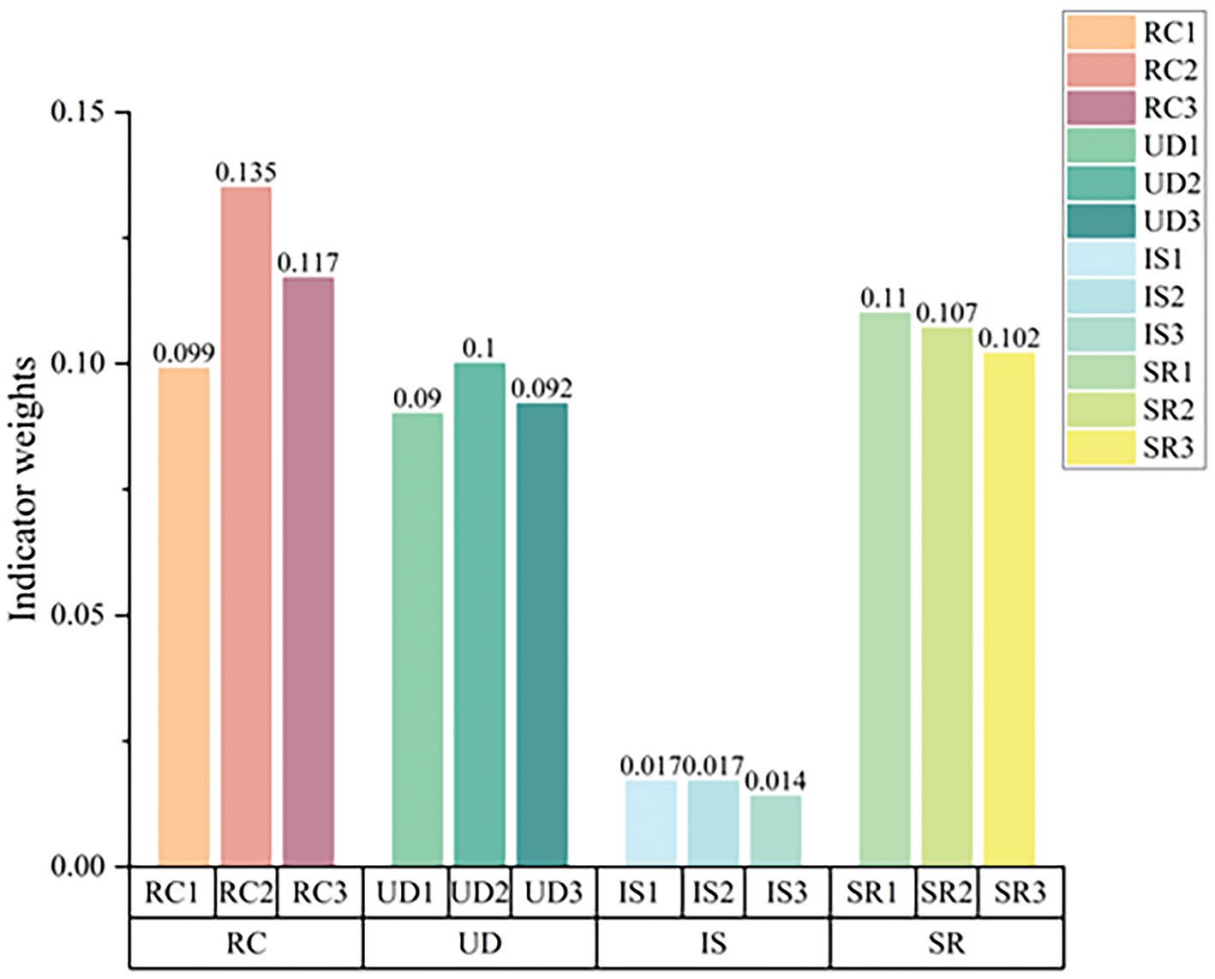
Weighting table of quality influencing factors.

Calculations using Eqs. (24)–(26) yielded the results of the weights of the factors affecting quality as shown in Fig. 9.

Figure 9 depicts the quality influencing factor weight table, the horizontal coordinates denote the quality influencing factors, and the vertical coordinates denote the influencing factor weight values. For instance, the tightness (RC1) quality indicator's weight value is 0.099, and other indicators are similar. The weight values for waterproofness (RC2) and elevation contrast (IS3) are shown in the Fig. 9. As can be seen, the weight value for waterproofness is the largest at 0.135 and the weight value for elevation contrast is the smallest at 0.014. This shows that Waterproofness has the greatest impact on quality while Elevation Control has the least impact.

**Figure 10:**
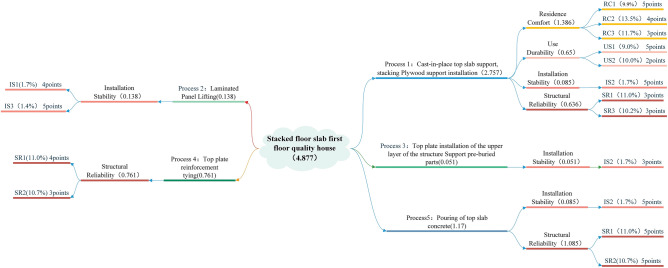
Scoring chart of quality house for laminated floor boards.

Equations (13), (14) are used to create a quality house to quantify the quality optimization contribution rate of prefabricated components on the basis of determining the weights of the quality influencing elements. Figure 10 displays the outcomes of quality optimization contribution rate scoring for laminated floor boards.

From Fig. 10, it can be seen that the scoring values of Z_11_, Z_12_,Z_13_ for the influencing factors RC1, RC2 and RC3 for the process 1 of laminated floor boards are 5, 4 and 3 points respectively, and the other indexes are similar. After the calculation of formula (13), the weight V_11_ of process 1 for residential comfort (RC) is 1.386, the weight V_12_ for service durability (US) is 0.650, the weight V_13_ for assembly stability (IC) is 0.085, and the weight V_14_ for structural reliability (SR) is 0.036, which is summarized to get the quality score of 2.757 for process 1, and the quality scores for the other processes are the same. The importance score of the stacked floor slab is 4.877 after summing the processes using Eq. (14). Using the quality house scoring process described above, when the quality optimization scores for the stacked floor factors are all perfect, the importance score of the stacked floor slab is 6.345. Using the importance score of the stacked floor slab as the numerator, and the denominator as the importance scores for the components assuming that the optimization scores are all perfect, the importance score for the other components is calculated similarly. Thus, the quality optimization rate for the laminated floor slab is calculated to be 76.864%, and the calculations for the other components are similar. Based on the calculation of the quality optimization rate of all components, the aggregated quality optimization rate SUMQ of all components is 29.677, and the quality optimization contribution rate Q of prefabricated components can be obtained by calculating the proportion of component quality optimization rate to the total component quality optimization rate SUMQ, as shown in Table 5.

**Table 5:** Contribution of different components to quality optimization.

Number of floors	Prefabricated components	Contribution to quality optimization (%)
First layer	Columns	2.29
	Beam	2.22
	Wall	2.59
	Boards	1.93
	Stairs	1.32
Second Floor	Columns	2.18
	Beam	1.91
	Wall	2.43
	Boards	1.80
	Stairs	1.09
Third to eleventh floor	Columns	2.01
	Beam	1.81
	Wall	2.37
	Boards	1.68
	Stairs	1.04

### Establishment of multi-objective optimization model

The following model represents the multi-objective cost, time, and carbon emission optimization of prefabricated component combination under several restrictions, including precast rate, quality optimization contribution rate, and anticipated value of each sub-objective.

Objective function:

25 $$ \left\{ \begin{gathered} \min C = \sum\nolimits_{i = 1}^{n} {C_{i1} X_{i} + } C_{i2} (1 - X_{i} ) \hfill \\ \min T = \sum\nolimits_{i = 1}^{n} {T_{i1} X_{i} + } T_{i2} (1 - X_{i} ) \hfill \\ \min E = \sum\nolimits_{i = 1}^{n} {E_{i1} X_{i} + } E_{i2} (1 - X_{i} ) \hfill \\ \end{gathered} \right. $$

Constraints:

26 $$ \left\{ \begin{gathered} Q = \sum\nolimits_{i = 1}^{n} {Q_{i1} X_{i} + } Q_{i2} (1 - X_{i} ) \hfill \\ A = \sum\nolimits_{i = 1}^{n} {A_{i1} X_{i} + } A_{i2} (1 - X_{i} ) \hfill \\ \end{gathered} \right. $$

27 $$ f(x) = \left\{ \begin{gathered} X_{i} = 1,\;{\text{The i - th component selects the cast - in - place construction process}} \hfill \\ X_{i} = 0,\;{\text{The i - th component is selected for the prefabricated construction process}} \hfill \\ \end{gathered} \right. $$

28 $$ C \le C^{*} ,\quad T \le T^{*} ,\quad E \le E^{*} ,\quad Q^{*} \le Q,\quad A^{*} \le A $$

where C* is the maximum threshold of cost, i.e., the contract price of the project; T* is the maximum threshold of duration, i.e., the target duration of the project; E* is the carbon emission limit of the project; Q* is the minimum quality optimization rate of the project, and A* is the minimum value of precast rate.

The precast rate constraint in Eq. (28) is equal to 50% according to the “Evaluation Standard for Prefabricated Buildings” (DBJT 15-163-2019) of Guangdong Province^83^, where the project is located. The precast rate of prefabricated structures is required to be no less than 50%. According to the project's bid documents, the contract price is set at C* = 540 × 10^4^ yuan, and the intended construction time is T* = 430. Set the carbon emission cap E* = 1730 t and the quality optimization contribution rate Q* = 60 in accordance with the real case scenario by utilizing the project of the same scale as a guide.

### Analysis of optimization results

**Figure 11:**
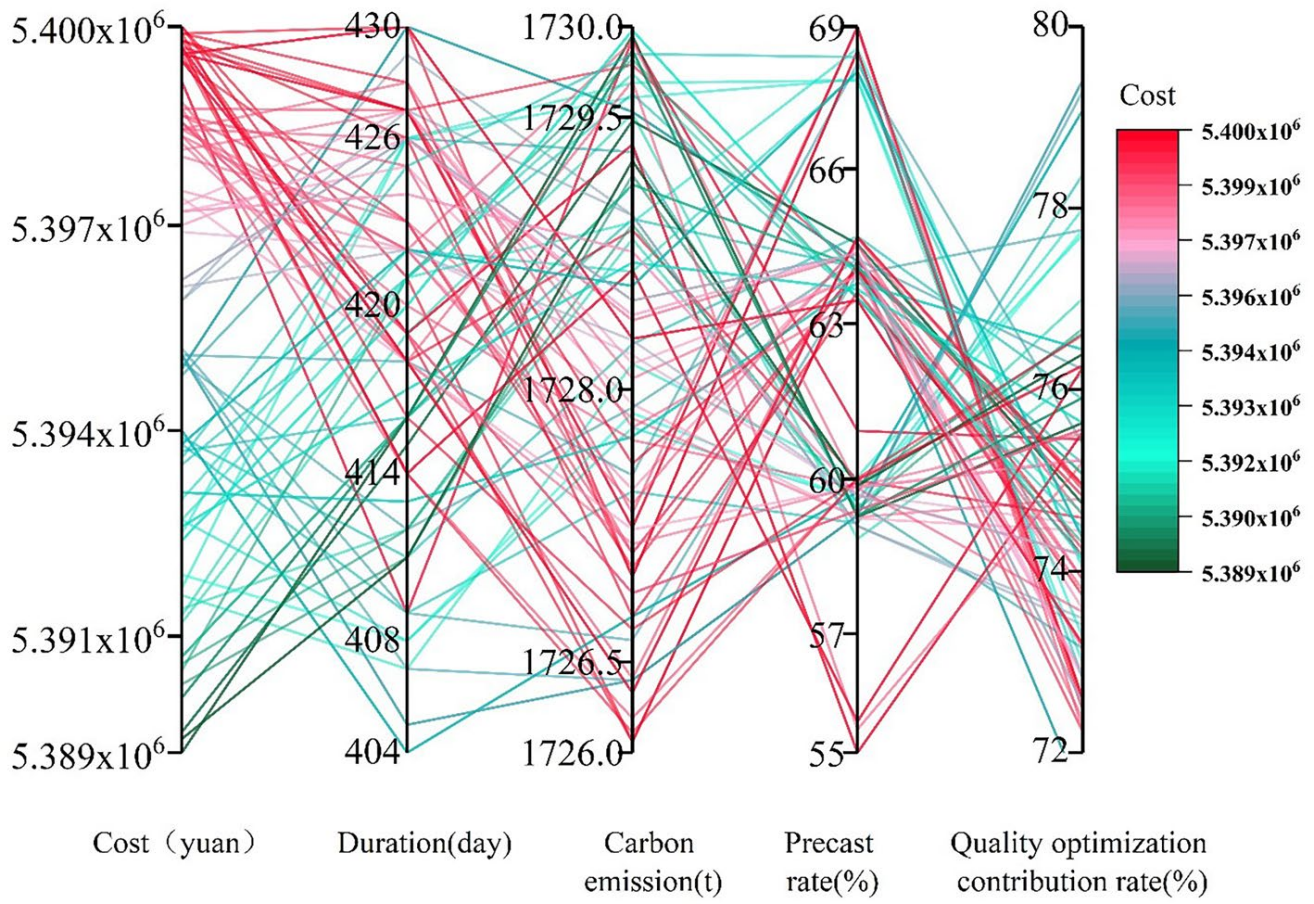
Pareto solution set diagram.

In order to solve the multi-objective optimization model Eq. (27) for the combination of prefabricated components, this research is built on the Matlab. R2019b platform (https://ww2.mathworks.cn/products/matlab.html) and uses NSGA-II. When the number of populations (N) is 100, the Pareto solution set of 100 prefabricated components will be obtained, and the number of iteration generations is 200, and the run to get the Pareto solution set graph of the prefabricated components is shown in Fig. 11, set the crossover probability of NSGA-II 0.8, variance probability 0.05, and the number of populations is related to the Pareto solution set of prefabricated components.

**Figure 12:**
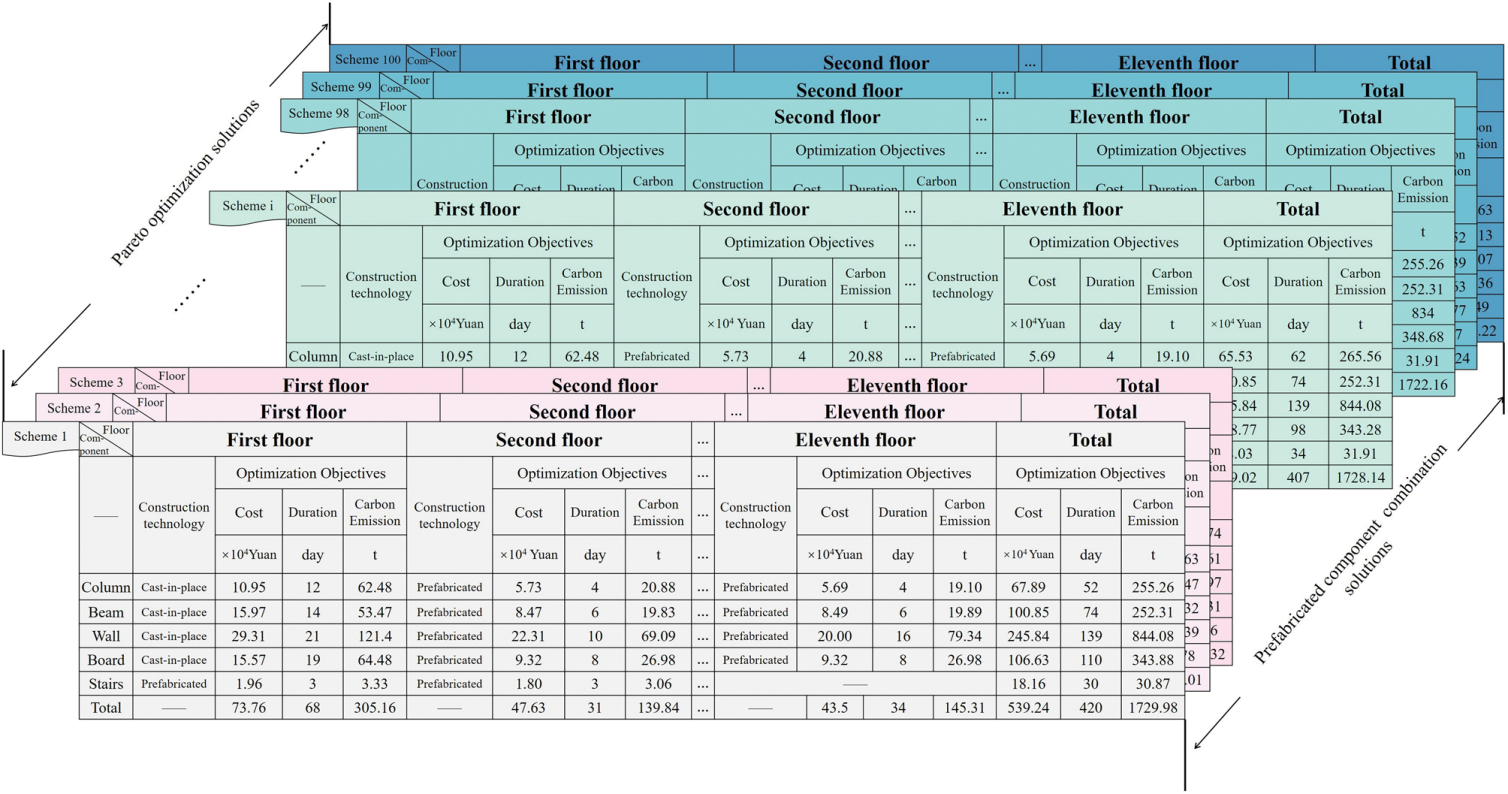
Prefabricated component combination solution.

This Fig. 11 displays the Pareto solution set of the outcomes of solving multi-objective optimization using NSGA-II. The horizontal axes represent the optimization objectives Cost, Duration, Carbon emission, constraints Precast rate, Quality optimization contribution rate, and the vertical coordinates consist of interval values for each optimization objective and constraints, such as: The interval value of Cost is 5.389 × 10^6^–5.40 × 10^6^ yuan, and the other values are shown in Fig. 11. The graph has 100 curves, each of which represents a Pareto optimal solution, for instance, where the cost is 5.399 × 10^6^ yuan, the duration is 419 days, the amount of carbon emission is 1726.06 t, the precast rate is 64.21%, and the contribution rate for quality improvement is 74.95%. The image shows that there is a significant variation in the curve of the parallel axis of the Pareto solution set, which suggests that the optimization goals of prefabricated component combinations are in conflict with one another and are constrained by one another. The trend of the curves shows that the precast rate will be relatively high when the Pareto optimal solution has a high cost, but the duration, carbon emission, and quality optimization contribution rate will be relatively low, and vice versa. This shows that there is a negative correlation between the cost of the Pareto optimal solution and the duration, carbon emission and quality optimization contribution rate, while there is a positive correlation with the precast rate. The main reason is that in order to optimize construction period, carbon emissions, and quality, it is often necessary to choose more expensive prefabricated components rather than cast-in-place components, which results in higher costs and prefabrication rates for the building. This indicates that there are trade-offs and balances between the various objectives, and selecting the optimal prefabricated component combination requires achieving a balance and optimal solution across different objectives. Each optimal solution in the set of Pareto solutions corresponds to a set of prefabricated component combination solutions, as shown in Fig. 12.

The 100 prefabricated component combination solutions are shown in Fig. 12, along with the results of choosing the construction methods for the columns, beams, walls, boards, stairs, and other components, as well as the overall cost, duration, carbon emission, precast rate, and quality optimization contribution rate of the corresponding solutions. These results were obtained using NSGA-II solving. Taking Solution 1 as an example, cast-in-place columns are selected for the first floor, and the cost of cast-in-place columns is 10.95 × 10^4^ yuan, the duration is 12 days, and the carbon emission is 62.48 t, and the selection of components for each floor is followed by analogy, so that the total cost of the final Solution 1 is 539.24 × 10^4^ yuan, the duration is 420 days, the carbon emission is 1730 t, and the precast rate is 63.94%, and the quality optimization contribution rate is 74.14%. The other programs are similar. Instead of an optimal solution, a set of optimal solutions is obtained by using NSGA-II. In order to find the optimal prefabricated component combinations, this paper evaluates the optimized prefabricated component combinations by optimizing the projection pursuit model through the simulated annealing algorithm.

**Figure 13:**
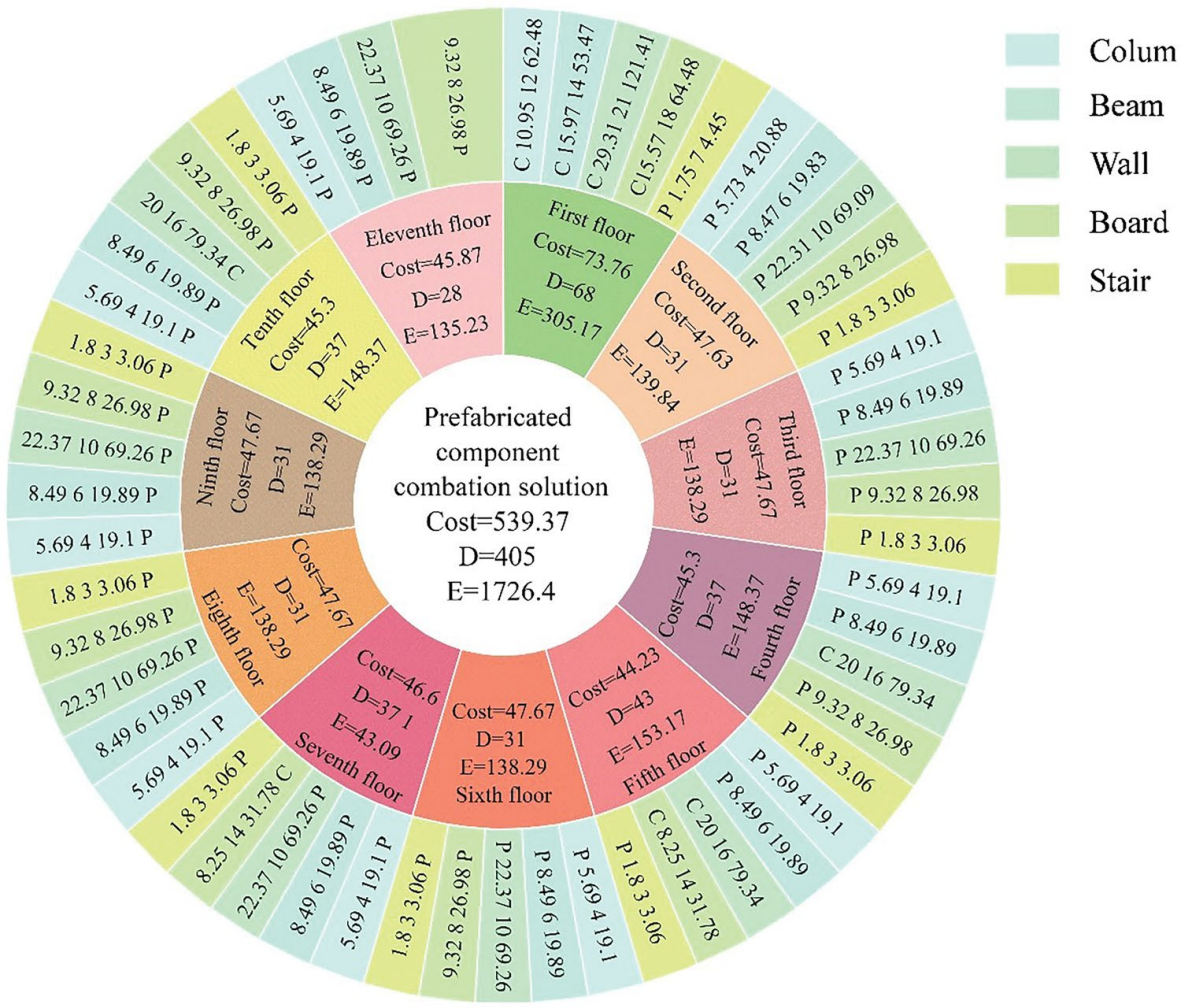
Prefabricated solution component selection chart.

The initial parameters of the simulated annealing algorithm were set: initial temperature T_0_ = 1000, termination temperature Tend = 0.0001, number of iterations N = 100, and the program was run using MATLAB. R2019b software (https://ww2.mathworks.cn/products/matlab.html). In the simulated annealing optimization projection pursuit dynamic model for solving, the best projection direction a* = (0.3097, 0.9951, 0.9976), the size of the optimal projection direction vector reflects the degree of influence of the evaluation indexes on the comprehensive evaluation, in which the carbon emissions of the evaluation results of the greatest impact, which is in line with China’s current “dual-carbon” goal of building energy saving. This is in line with the requirement of energy saving and emission reduction in buildings under the current “dual carbon” goal of China. Using the best projection direction to calculate the projection eigenvalue of the prefabricated component combination program, the maximum projection eigenvalue of the program is 2.0491, indicating that the program is optimal, and the selection of program components is shown in Fig. 13.

Figure 13 shows a multi-layer circular pie chart for the optimal solution component selection case, which is divided into three layers from inside to outside. The innermost layer is the case of the optimal solution, the cost of the optimal solution is 539.37 × 10^4^ yuan, the duration is 405 days, and the carbon emission is 1726.4 t. The middle layer is the case of the eleven floors of the building respectively, taking the first floor as an example, the total cost of the component selection of the first floor is 73.76 × 10^4^ yuan, the duration is 68 days, and the carbon emission is 305.17 t, and the other floors are similar. The outermost layer shows the selection of columns, beams, walls, boards, and stairs components for each floor, and different components are indicated by different colors. The selection of components for the first floor is cast-in-place columns, cast-in-place beams, cast-in-place boards, cast-in-place walls, and prefabricated staircases, in which the cost of cast-in-place columns is 10.95 × 10^4^ yuan, the duration is 12 days, and the carbon emission is 62.48 t, and the selection of components for other floors is similar. After analyzing the component selection of the optimal solution, it is concluded that the main reason for this optimal solution is the greater selection of prefabricated columns, prefabricated beams, and prefabricated stairs. These components provide higher cost, construction period, and carbon emission benefits compared to other components at different prefabrication rates. Additionally, prefabricated columns, beams, and stairs have various advantages in construction efficiency, quality control, space utilization, energy efficiency, and emission reduction. Therefore, for the component selection in the prefabricated building frame structure, prefabricated columns, beams, and stairs should be prioritized.

**Figure 14:**
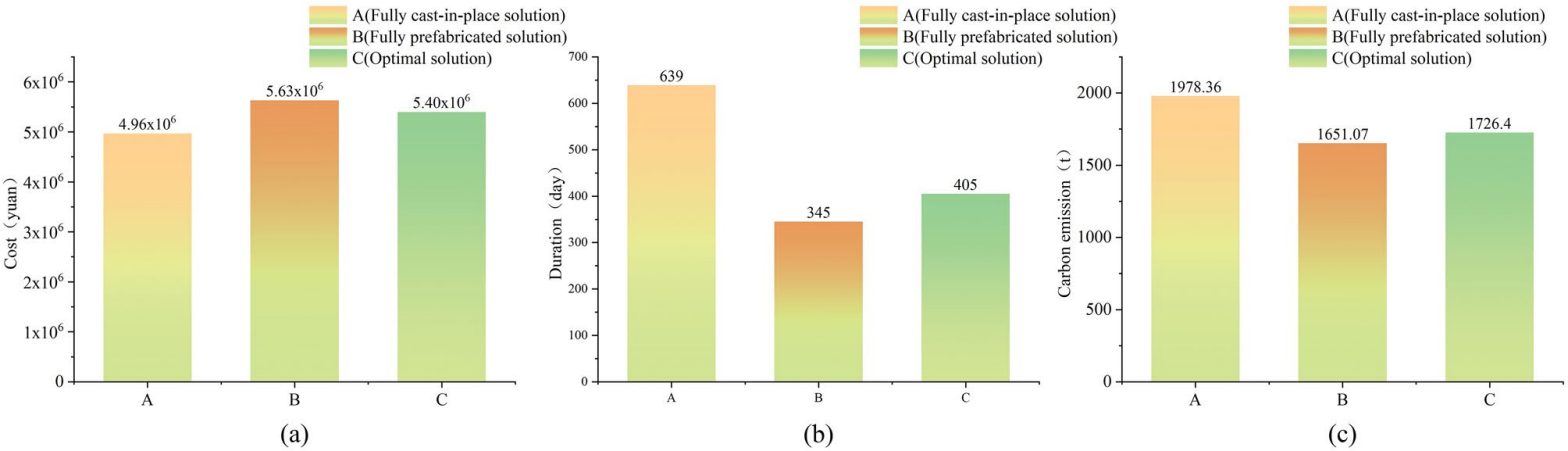
Comparison of evaluation program results.

Taking the all-cast-in-place and all-prefabricated solution as the base case, the optimal prefabricated component combination solution is studied in comparison with the base case to analyze the optimization effect of component combination, and the comparison results are shown in Fig. 14.

Figure 14 shows a comparative study of the optimal prefabricated component combination solution with the baseline case, where (a), (b), and (c) are the comparisons of cost, duration, and carbon emission, respectively, with Scenario A being the all-cast-in-place solution, Scenario B being the all-prefabricated solution, and Scenario C being the prefabricated solution with the optimal evaluation results. From Fig. 14, it can be seen that the optimal prefabricated component combination solution increases the cost by about 8.69% compared with the all-cast-in-place solution, but the duration decreases by about 36.62% and the carbon emission decreases by about 12.74%, which indicates that the optimal solution has greater advantages in carbon emission and duration compared with the all-cast-in-place solution, although it has a higher cost. The optimal prefabricated component combination solution increases the duration by 17.39% and the carbon emission by 4.56% compared with the fully prefabricated solution, but the cost decreases by 4.15%, which indicates that the optimal solution is less costly compared with the fully prefabricated solution, although it lacks the optimization degree in terms of carbon emission and duration. This is mainly due to the application of prefabricated components. Although the costs in production and transportation stages are higher for prefabricated components, they have distinct advantages in terms of construction period and carbon emissions. With the gradual development of prefabricated construction, more efficient production methods and standardized design will reduce the cost of prefabricated components, thereby gradually revealing the cost advantages of prefabricated construction. In summary, the prefabricated component combination obtained through multi-objective optimization has certain advantages compared to both fully cast-in-place and fully prefabricated schemes.

## Discussion

This method considers cost, duration, and carbon emissions as objective functions, and the construction process of cast-in-place and prefabricated components as variables. The constraints include prefabrication rate, quality optimization rate, and expected values of each sub-objective. NSGA-II is used as the tool to solve the multi-objective optimization model and obtain a set of optimal Pareto solutions for prefabricated component combinations. Based on the optimal Pareto solution set, the simulated annealing optimization projection tracing method is used to establish a multi-objective evaluation model to evaluate the solutions and select the optimal prefabricated component combination. An empirical study is conducted using an eleven-story frame structure building in Shenzhen, Guangdong Province, China as an example. The optimization results show that the prefabricated component combination solutions obtained through multi-objective optimization and evaluation have advantages over fully cast-in-place and fully prefabricated solutions. The Pareto solution set can provide effective support for decision-making in engineering projects, and stakeholders can select the most reasonable prefabricated component combination based on their own circumstances and construction needs.

### Contrast analysis

This study differs from traditional engineering projects that focus solely on multi-objective optimization in terms of construction period, cost, and quality. In recent years, more and more scholars have begun to focus on energy efficiency and low carbon in buildings, approaching research from various perspectives. Liang et al. constructed a multi-objective optimization mathematical model based on the combination of project construction execution modes, showing a 7.36% reduction in component carbon emissions^84^. Xue selected insulation thickness, window type, window-to-wall ratio, overhang depth, and building orientation as design variables and proposed a simulation-based multi-objective optimization model. Compared to the initial design, the minimum possible reduction in lifecycle carbon emissions was 13.5%^85^. Yang conducted multi-objective optimization based on a stacking model and NSGA-III algorithm for material thickness, window-to-wall ratio, and solar heat gain coefficient. The optimized building operational energy consumption decreased by 45.38%, and carbon emissions were increased by 10.75 kg^86^. This paper takes the prefabricated component combination as the optimization target, providing a new research perspective. The optimal solution obtained in this study reduces carbon emissions by 12.74% compared to the fully cast-in-place scheme.

This reduction in carbon emissions is relatively significant compared to other research results, offering an effective pathway for buildings to transition from cast-in-place construction to prefabricated construction and sustainable development. However, the multi-objective optimization model constructed by Liang et al. resulted in a 1.55% reduction in carbon emissions and a 3.52% reduction in construction period^84^. Xue’s energy simulation model with mixed ventilation and dimming control optimization could potentially reduce costs by 10.9–18.9% while lowering carbon emissions^85^. In contrast, the study on prefabricated component combinations in this paper shows a slight increase in costs when reducing carbon emissions and construction period. This is mainly due to the high cost of prefabricated components, high construction efficiency, and the characteristics of energy-saving and emission reduction. Additionally, the costs considered in this study are construction costs and do not account for the impact of reduced construction period and carbon emissions on costs. When considering the cost of construction period and carbon emissions, the optimized prefabricated component combination can reduce costs, construction period, and carbon emissions compared to cast-in-place construction.

### Advantages and limitations

This study has two main improvements. Firstly, it innovatively incorporates carbon emission objectives into traditional engineering management multi-objective optimization. By considering prefabricated components as the research object, the study obtains optimal Pareto solutions, allowing stakeholders to select the most reasonable prefabricated component combinations based on their own circumstances and construction needs. This optimization approach enables the rational selection of prefabricated components and has practical value for the scientific and sustainable development research of modular construction. Secondly, this study combines NSGA-II and the simulated annealing optimization projection tracing model and applies them to the multi-objective optimization problem of prefabricated component combinations. This innovative approach effectively solves the multi-objective optimization problem of prefabricated component combinations, improving the design efficiency and the quality of optimization results. Moreover, this method can also be applied to the solution of other multi-objective optimization problems, demonstrating its generality and potential for further application. However, the method proposed in this study is merely an exploratory attempt and still has certain limitations: (1) The cost mentioned in this study mainly refers to the construction and installation cost, neglecting the impact of duration and carbon emissions on cost. In practical engineering projects, the cost should be comprehensive, including construction and installation cost, duration cost, and carbon emissions cost. Calculating the comprehensive cost of prefabricated components can better assess their economic and rationality under the current context of building energy efficiency, thus making more economically and sustainable decisions. (2) The proposed method is a deepening design of component construction process selection based on the preliminary determination of the building structure. It assumes that choosing between cast-in-place or prefabricated building methods will not affect the structural forces. However, in real engineering projects, the choice of cast-in-place or prefabricated construction methods can have an impact on the structural forces and deformations. These two points are directions for further research in order to make the proposed method more scientifically and logically sound.

### Reliability and universality

This project proposes a multi-objective optimization method for prefabricated component combinations based on cost, duration, and carbon emissions, and conducts an empirical study using an eleven-story frame structure building in Shenzhen, Guangdong Province, China as an example. This method has strong universality and can be applied to prefabricated building projects in different countries. Based on the multi-objective optimization and evaluation model for prefabricated component combinations constructed in this study, stakeholders can collect actual engineering parameters of the project and incorporate them into the model. Then, artificial intelligence algorithms can be used to solve the multi-objective problem and choose the optimal solution based on the results obtained from the Pareto solution set, providing reference for the sustainable development of local prefabricated building. However, the constraint of quality optimization rate in this method needs to be determined by constructing a structural equation model based on the collection of survey questionnaire data. This requires selecting samples that are representative of the local level of prefabricated building development and regional conditions to ensure the scientific and reliable analysis results. Additionally, the method proposed in this paper mainly applies to concrete structures in prefabricated building and is not applicable to wood structures and steel structures.

### Research significance

Sustainable development has become an inevitable path for the future development and transformation of the construction industry in China. As the main mode of construction for sustainable development, prefabricated building has become an important means for the government to promote the economic structural transformation of the construction industry and its sustainable development. Incorporating carbon emission objectives into the multi-objective optimization of engineering project management is a new management concept in the context of energy conservation and emission reduction. This can maximize cost and duration savings while minimizing carbon emissions from buildings, under the condition of meeting the requirements for prefabrication rate. It greatly promotes the rationality of prefabricated component selection and the enthusiasm for the development of prefabricated building. The multi-objective optimization model and solution algorithm proposed in this study can be applied to guide engineering practice, providing stakeholders with the optimal prefabricated component combinations. This allows for the achievement of good social, economic, and environmental benefits after the project is completed, while considering sustainability in the comprehensive optimization management of engineering projects, making it more suitable for modern management optimization.

## Conclusions and recommendations

### Conclusions

This paper establishes a multi-objective optimization model based on cost, duration, and carbon emissions, considering the constraints of prefabrication rate, quality optimization contribution rate, and expected values of sub-objectives. The NSGA-II algorithm is applied to solve the multi-objective optimization model. Additionally, the simulated annealing algorithm is used to optimize the projection tracing dynamic model to evaluate the prefabricated component combinations. The empirical study is conducted using an eleven-story frame structure building in Shenzhen, Guangdong Province, China. The conclusions are as follows:

1. The optimal solution, compared to the fully cast-in-place solution, has an increase in cost of approximately 8.69%, but a decrease in duration of about 36.62% and a reduction in carbon emissions of about 12.74%. Compared to the fully prefabricated solution, the optimal solution has an increase in duration of 17.39%, an increase in carbon emissions of 4.56%, but a decrease in cost of 4.15%. This indicates that the prefabricated component combination solution obtained through multi-objective optimization has certain advantages over both fully cast-in-place and fully prefabricated solutions.
2. There is a negative correlation between the cost of prefabricated component combinations and the factors of duration, carbon emissions, and quality optimization contribution rate. However, there is a positive correlation between the cost and the prefabrication rate.
3. When using the simulated annealing algorithm to optimize the projection tracing method for comprehensive evaluation of the solutions, it is found that carbon emissions have the greatest impact on the evaluation results based on the magnitude of the optimal projection direction vector. This aligns with the current requirements for energy conservation and emission reduction in the building sector in China under the “dual carbon” goals.

### Recommendations

Based on the research in this paper, the following suggestions are proposed to promote the adoption of prefabricated concrete construction in a more scientific and rational manner:

1. The research in this paper demonstrates the importance of selecting the appropriate prefabricated component combinations to achieve good social, economic, and environmental benefits after project completion. Therefore, decision-makers need to discuss their own needs and conditions during the early decision-making stage of a construction project. Factors such as project positioning and the conditions of prefabricated component production and transportation in the project location should be taken into account to select the most suitable prefabricated component combination solution.
2. When selecting prefabricated components, consideration should be given to components that provide significant savings in duration and carbon emissions per unit cost. In the optimal solution, prefabricated columns, beams, and stairs are selected more frequently, indicating their clear advantages in the selection of component combinations for frame structures. Therefore, these components can be prioritized in component selection.
3. The carbon emission objective has the greatest impact on the evaluation results of the prefabricated component combination solutions. In the context of energy conservation and emission reduction in the building sector, stakeholders should pay more attention to managing the carbon emissions throughout the entire lifecycle of prefabricated components. Optimizing production processes to reduce the carbon emissions of prefabricated components is crucial. When selecting components, choose prefabricated components with lower overall carbon emissions to achieve the low-carbon and environmental goals of buildings.

## Data Availability

The data underlying the results presented in the study are included within the manuscript.
